# The vacuolar transporters MaMATE11 and MaMATE14 affect blue flower coloration in grape hyacinth (*Muscari*)

**DOI:** 10.1093/hr/uhaf270

**Published:** 2025-10-17

**Authors:** Xiaoyun Cao, Jingwen Xie, Xuelan Gao, Wanqi Pan, Jiaxin Gong, Lingjuan Du

**Affiliations:** College of Landscape Architecture and Arts, Northwest A&F University, Yangling, Shaanxi 712100, China; State Key Laboratory of Crop Stress Resistance and High-Efficiency Production, Northwest A&F University, Yangling, Shaanxi 712100, China; Key Laboratory of Horticultural Plant Biology and Germplasm Innovation in Northwest China, Ministry of Agriculture, Yangling, Shaanxi 712100, China; College of Landscape Architecture and Arts, Northwest A&F University, Yangling, Shaanxi 712100, China; State Key Laboratory of Crop Stress Resistance and High-Efficiency Production, Northwest A&F University, Yangling, Shaanxi 712100, China; Key Laboratory of Horticultural Plant Biology and Germplasm Innovation in Northwest China, Ministry of Agriculture, Yangling, Shaanxi 712100, China; College of Landscape Architecture and Arts, Northwest A&F University, Yangling, Shaanxi 712100, China; State Key Laboratory of Crop Stress Resistance and High-Efficiency Production, Northwest A&F University, Yangling, Shaanxi 712100, China; Key Laboratory of Horticultural Plant Biology and Germplasm Innovation in Northwest China, Ministry of Agriculture, Yangling, Shaanxi 712100, China; College of Landscape Architecture and Arts, Northwest A&F University, Yangling, Shaanxi 712100, China; State Key Laboratory of Crop Stress Resistance and High-Efficiency Production, Northwest A&F University, Yangling, Shaanxi 712100, China; Key Laboratory of Horticultural Plant Biology and Germplasm Innovation in Northwest China, Ministry of Agriculture, Yangling, Shaanxi 712100, China; College of Landscape Architecture and Arts, Northwest A&F University, Yangling, Shaanxi 712100, China; State Key Laboratory of Crop Stress Resistance and High-Efficiency Production, Northwest A&F University, Yangling, Shaanxi 712100, China; Key Laboratory of Horticultural Plant Biology and Germplasm Innovation in Northwest China, Ministry of Agriculture, Yangling, Shaanxi 712100, China; College of Landscape Architecture and Arts, Northwest A&F University, Yangling, Shaanxi 712100, China; State Key Laboratory of Crop Stress Resistance and High-Efficiency Production, Northwest A&F University, Yangling, Shaanxi 712100, China; Key Laboratory of Horticultural Plant Biology and Germplasm Innovation in Northwest China, Ministry of Agriculture, Yangling, Shaanxi 712100, China

## Abstract

The development of blue flower coloration involves the biosynthesis, transport, and accumulation of flavonoids in petal epidermal cells. Although the mechanisms of flavonoid biosynthesis and regulation are well understood, much less is known about the molecular basis of vacuolar anthocyanin/flavonoid sequestration. Here, we identified two tonoplast-localized MATE transporters, MaMATE11 and MaMATE14, that participate in flavonoid transport and influence the blue color of grape hyacinth petals. *In vitro* transport experiments revealed that both proteins transported a range of flavonoid substrates, with a preference for malonylated anthocyanins, but differed in their substrate specificity and kinetic parameters. Both *MaMATE11* and *MaMATE14* could complement the anthocyanin-deficient phenotype of the *Arabidopsis AtDTX35* mutant, and silencing of either gene by RNA interference significantly reduced anthocyanin accumulation in petals of grape hyacinth. Expression of *MaMATE11* and *MaMATE14* was directly activated by the anthocyanin-biosynthesis-related transcription factors MaMybA and MaAN2, respectively, establishing a coordinated anthocyanin synthesis–transport module. These findings provide insight into mechanisms of floral coloration and flavonoid translocation in blue-pigmented species and identify valuable target genes for molecular breeding of ornamental flower colors.

## Introduction

Flavonoids are a major class of plant-specific metabolites with diverse physiological functions [1]. They are biosynthesized at the endoplasmic reticulum (ER) membrane and subsequently transported to vacuoles or other cellular compartments for storage [2]. One class of flavonoids, the anthocyanins, act as visual cues to attract pollinators and seed distributors to flowers and fruits [3]. Flavonoids are produced by the phenylpropanoid biosynthetic pathway and then modified through glycosylation, acylation, and methylation reactions [4]. The specific modifications that decorate flavonoids may determine which membrane proteins mediate their vacuolar transport, ultimately affecting their accumulation in plants [5, 6]. A previous study suggested that vacuolar sequestration is a prerequisite for flavonoid biosynthesis and a crucial process in flavonoid metabolism [7]. The vacuolar compartmentalization of flavonoids serves three critical functions: (i) it enables their long-term storage, (ii) it provides the acidic environment necessary for specific glycosylation/acylation reactions, and (iii) it facilitates substrate access to vacuolar-localized modifying enzymes (glycosyltransferases and acyltransferases) [2]. Despite decades of research, much less is known about the molecular mechanisms of flavonoid vacuolar transport than about flavonoid biosynthetic pathways.

Transport of flavonoids into the vacuole is thought to occur in two sequential steps: flavonoids are first delivered from the ER surface to the tonoplast, then transported across the tonoplast into the vacuole [8, 9]. Two distinct—but not necessarily mutually exclusive—models have been proposed to explain the transport of flavonoids from their sites of biosynthesis to the central vacuole: vesicle-mediated and transporter-mediated transport [8, 10]. The vesicle-mediated pathway uses autophagosomes (macro−/microautophagy) for flavonoid delivery [11–13], whereas the transporter-mediated pathway involves glutathione-S-transferase (GST)-escorted flavonoid trafficking to ABCC/MATE transporters on the tonoplast [4, 6]. Notably, GSTs exhibit dual functionality: recent evidence confirms that they catalyze a step in anthocyanidin biosynthesis [14] as well as mediating compartment-specific anthocyanin transport, and GST suppression leads to accumulation of anthocyanins in small vesicles, whereas MATE inhibition causes anthocyanin accumulation mainly in the vacuole [15].

Several lines of evidence indicate that the ABCC transporters ZmMRP3 in maize (*Zea mays*) [16], ABCC1 in grapevine (*Vitis vinifera*) [4], and AtABCC2 in *Arabidopsis* [17] participate in vacuolar sequestration of anthocyanins. ABC transporters are primary active transporters that use energy derived from ATP hydrolysis, whereas MATE transporters are secondary active antiporters driven by the H^+^ electrochemical gradient across the tonoplast [2]. Numerous MATE transporters have also been shown to transport different classes of flavonoids into vacuoles; examples include the transport of anthocyanins by AM1 and AM3 in grapevine [6, 15], MtMATE2 in *Medicago truncatula* [18], LhDTX35 in *Lilium* [19], and PhMATE1 in *Petunia* [20]; the transport of proanthocyanins by TT12 in *Arabidopsis* [7], MtMATE1 in *M. truncatula* [21], and DkDTX5 in persimmon (*Diospyros kaki*) [22]; the transport of flavonols by NtMATE21 and NtMATE22 in *Nicotiana tabacum* [23]; and the transport of isoflavones by GmMATE1 and GmMATE2 in soybean [24]. Despite these reports, fundamental questions about flavonoid transport mechanisms remain, including how vacuolar transporters differ in their substrate specificity and transport kinetics, and how their substrate preferences are influenced by specific flavonoid side-chain modifications.

Previous studies have shown that flavonoid biosynthesis is regulated at the transcriptional level by R2R3 MYB–bHLH–WD40 (MBW) transcription factor complexes [25]. Within these complexes, the R2R3 MYB proteins are the primary determinants that regulate different branches of flavonoid biosynthesis [26]. Recent evidence suggests that R2R3 MYB proteins also regulate the expression of genes linked to flavonoid transport, such as *GSTs* [27, 28], thereby influencing the delivery of cytoplasmic flavonoids to the tonoplast. MdMYB1/10 and AtPAP1, master regulators of anthocyanin synthesis, transcriptionally activate genes encoding proton pumps (*MdVHAs*/*MdVHP*) and an anthocyanin transporter (*MdMATE-LIKE1*) in apple [29], and NtMYB12 transactivates the flavonol transporter genes *NtMATE21* and *NtMATE22* in tobacco [10]. Nonetheless, it is not clear whether different regulatory proteins selectively target transporters with distinct substrate specificities and physiological functions, and the mechanisms that regulate flavonoid transport remain to be clarified.

Flower color is among the most essential characteristics of ornamental plants. Considered by many to be the most intriguing and romantic flower color, blue arises primarily from the synthesis of glycosylated, acylated, or methylated delphinidin-type anthocyanins [30]. The mechanisms responsible for blue floral pigmentation are well characterized and include higher vacuolar pH, synthesis of aromatic-acylated anthocyanins, complexation of anthocyanins with metal ions, and stacking of anthocyanins with copigments [30, 31]. However, little is known about the transport of anthocyanins into the vacuoles of blue flowers, including the identities of the specific transporters involved.

Grape hyacinth (*Muscari* spp.) is widely used in garden landscaping because of its distinctive violet-blue flowers. In previous work, we found that these violet-blue flowers contained mainly simple delphinidin-type derivatives, particularly petunidin-3-*O*-glucoside, malvidin-3-*O*-glucoside, and delphinidin-3-*O*-glucoside. By contrast, white flowers of grape hyacinth contained mainly pelargonidin/cyanidin derivatives with acyl moieties such as pelargonidin-3-*O*-sinapylglucoside-5-*O*-arabinoside and cyanidin-3-*O*-(*p*-coumaroyl)glucoside-5-*O*-malonylglucoside [32]. Two anthocyanin-related R2R3 MYB transcriptional activators (MaMybA and MaAN2) and an R3 MYB transcriptional repressor (MaMYBx) have also been identified in grape hyacinth [26, 33, 34].

In the present work, we identified two vacuolar transporters, MaMATE11 and MaMATE14, that participate in flavonoid transport and influence the blue color of grape hyacinth petals. Although they differ in substrate affinities and transport kinetics, both can mediate the transport of a broad spectrum of flavonoid substrates and show a preference for malonylated anthocyanins. Silencing of *MaMATE11* or *MaMATE14* by RNA interference blocked anthocyanin accumulation in grape hyacinth petals, and their expression complemented the anthocyanin-deficient phenotype of the *Arabidopsis AtDTX35* mutant. Multiple assays demonstrated that *MaMATE11* and *MaMATE14* are regulated by the anthocyanin-related R2R3 MYB activators MaMybA and MaAN2, respectively, in conjunction with MabHLH1 and the repressor MaMYBx. These results advance our understanding of the transcriptional regulation of petal color and flavonoid transport in blue flowers.

## Results

### Identification of the key MATE genes in grape hyacinth

**Figure 1:**
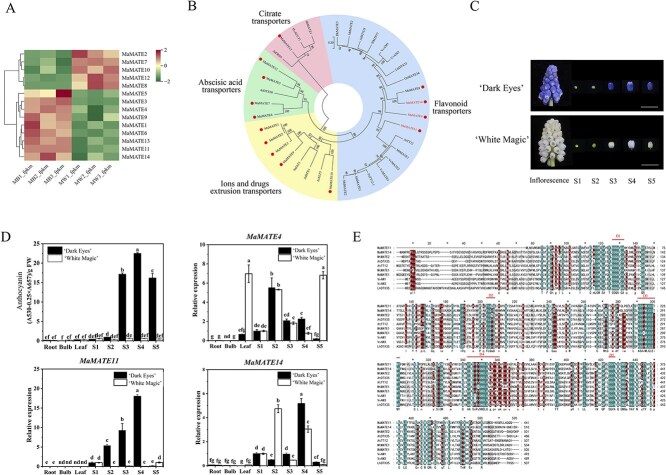
Identification of MATE transporters probably involved in flavonoid transport in grape hyacinth blue-flowers. (A) Heatmap of transcript levels for *MATE* genes in flowers of *M. aucheri* ‘Dark Eyes’ and ‘White Magic’. MB1_fpkm, MB2_fpkm, and MB3_fpkm represent the FPKM values of three biological replicates of ‘Dark Eyes’, MW1_fpkm, MW2_fpkm, and MW3_fpkm represent the FPKM values of three biological replicates of ‘White Magic’. The significant DEGs were screened out based on the log2FC values ≧ 1 and FDR < 0.05. (B) Phylogenetic tree of MATE proteins from grape hyacinth and other MATEs with known functions. The nodes display bootstrap values based on 1000 replicates. Different subfamilies are highlighted using different colors (flavonoid transporters in blue, ions and drugs extrusion transporters in yellow, abscisic acid transporters in green, and citrate transporters in pink). MATE proteins in grape hyacinth are marked by red-colored dots. The GenBank accession numbers are listed in Table S2. (C) Inflorescence and petals of five flower developmental stages (S1–S5) of ‘Dark Eyes’ and ‘White Magic’. Scale bar: 1 cm. (D) Total anthocyanin content and expression profile of *MaMATE4*, *MaMATE11*, and *MaMATE14* in root, bulb, leaf, and petals at S1–S5 in ‘Dark Eyes’ and ‘White Magic’. FW: Fresh weight. The data are expressed as means ± SD (*n* = 3), and distinct letters on the bars denote statistically significant differences (*P* < 0.05, Tukey’s HSD test). (E) Sequence alignment of MaMATE11 and MaMATE14 and characterized flavonoid MATE transporters. Red lines above the aligned sequences indicate the conserved protein regions (D1–D5).

To identify the key MATE genes potentially associated with the blue-colored flower phenotype, RNA-Seq analysis was performed on petal samples at Stage 4 of floral development from ‘Dark Eyes’ and ‘White Magic’ (Fig. 1C). The phenotypes of these two cultivars are shown in Fig. S1. Fourteen differentially expressed *MATE* transporters (*MaMATE1* to *MaMATE14*) are screened (Fig. 1A). Among them, *MaMATE1*, *MaMATE3*, *MaMATE4*, *MaMATE5*, *MaMATE6*, *MaMATE9*, *MaMATE11*, *MaMATE13*, and *MaMATE14* had a relatively high FPKM in ‘Dark Eyes’. To further screen the MATE transporters involved in flavonoid transport, a phylogenetic analysis of 14 MaMATE transporters (MaMATE1 to MaMATE14) with other known and putative MATE transporters involved in the transport of flavonoids, citrate, and abscisic acid, or ions and drug extrusion, was carried out [35]. The result showed that four MaMATE transporters (MaMATE4, MaMATE8, MaMATE11, and MaMATE14) fell into the group of flavonoid transport proteins (Fig. 1B). Collectively, *MaMATE4*, *MaMATE11*, and *MaMATE14* were identified as the putative *MATE* genes implicated in flavonoid transport during grape hyacinth blue flower formation. To further determine whether the expressions of *MaMATE4*, *MaMATE11*, and *MaMATE14* were linked to the blue petal phenotype and the anthocyanin content of petals in ‘Dark Eyes’ and ‘White Magic’, the abundance of transcripts was determined by quantitative real-time (qRT-) PCR, and the total anthocyanin contents in root, bulb, leaf, and petals at five floral developmental stages (Stages 1–5) in ‘Dark Eyes’ and ‘White Magic’ were analyzed by UV-Visible Spectrophotometer. As shown in Fig. 1D, the expression of *MaMATE11* approximately matched the anthocyanin distribution in ‘Dark Eyes’ and ‘White Magic’, while the expressions of *MaMATE4* and *MaMATE14* did not. MaMATE4 transcript levels were high in non-anthocyanin-pigmented tissues (leaf). MaMATE14 transcript levels were notably elevated in ‘White Magic’s Stage 2. These findings suggested that *MaMATE11* expression levels may be closely related to petal anthocyanin accumulation in ‘Dark Eyes’, whereas *MaMATE14* may be involved in both petal anthocyanin accumulation in ‘Dark Eyes’ and the accumulation of other flavonoids in white petals in ‘White Magic’. Therefore, *MaMATE11* and *MaMATE14* were identified as potential candidate genes for the blue flower phenotype. We cloned the cDNA sequences of *MaMATE11* and *MaMATE14* from ‘Dark Eyes’, which encoded the putative MATE proteins of 441 and 510 amino acids, respectively (GenBank accession numbers: OQ185274 and OQ185275) (Fig. S2 and S3). Further sequence alignment demonstrated that these two MATE proteins exhibited 52.31% identity to each other and shared the amino acid sequence identity (44.84%–53.00%) with other flavonoid MATE transporters, such as AtTT12, MtMATE1/2, etc. (Fig. 1E). The prediction of transmembrane domains suggested 10 and 12 putative transmembrane segments for MaMATE11 and MaMATE14, respectively (Fig. S4). It suggested that these two proteins were flavonoid MATE transporters.

### MaMATE11 and MaMATE14 localize to the tonoplast

**Figure 2:**
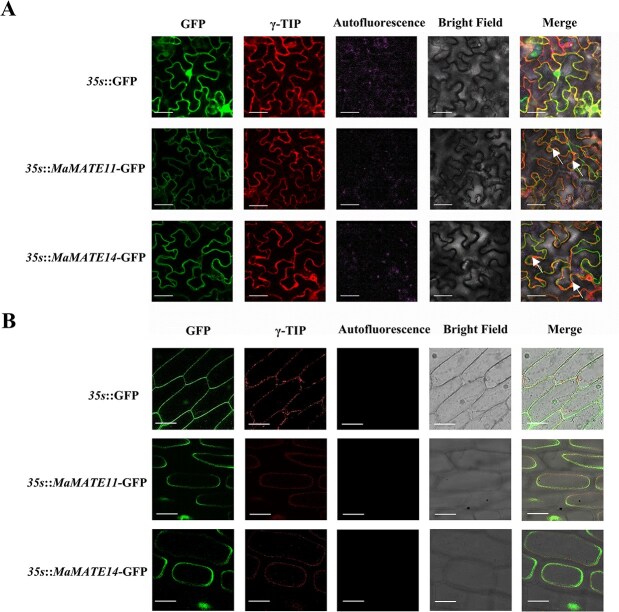
Subcellular localization of MaMATE11 and MaMATE14 proteins in (A) tobacco leaves and (B) purple onion bulb epidermal cells (without anthocyanins). γ-TIP-mCherry was used as a tonoplast marker. White arrow represented GFP fluorescence colocalized with the fluorescence signal of the tonoplast marker γ-TIP on the tonoplast. Bars, 25 μm.

The cellular localization patterns of MaMATE11 and MaMATE14 were examined by transiently expressing *35s::MaMATE11*-green fluorescent protein (GFP) and *35s*::*MaMATE14*-GFP in *Nicotiana benthamiana* leaves through *Agrobacterium* infiltration. The *35s::*GFP vector (GFP driven by the 35s promoter) served as the control. To confirm tonoplast targeting, we simultaneously expressed a mCherry-fused γ-TIP marker [36]. Tobacco epidermal cells expressing *35s*::*MaMATE11*-GFP and *35s::MaMATE14*-GFP exhibited intracellular membrane-bound GFP fluorescence and colocalized with the tonoplast marker γ-TIP on the tonoplast (Fig. 2A). However, GFP fluorescence of the control was observed throughout the cell (Fig. 2A). To validate the localization of MaMATE11 and MaMATE14, we extended the localization analysis of MaMATE11 and MaMATE14 in purple onion bulb epidermal cells by the *Agrobacterium*-mediated transformation method. The result showed that the GFP signals of *35s::MaMATE11*-GFP and *35s::MaMATE14*-GFP colocalized with the signals of the tonoplast marker γ-TIP in purple onion bulb epidermal cells (Fig. 2B, Fig. S5). Taken together, our results indicate that MaMATE11 and MaMATE14 localize to the tonoplast in *N. benthamiana* plants, which is supported by MaMATE11 and MaMATE14 tonoplast localization in purple onion epidermal cells.

### MaMATE11 and MaMATE14 are involved in anthocyanin transport

**Figure 3:**
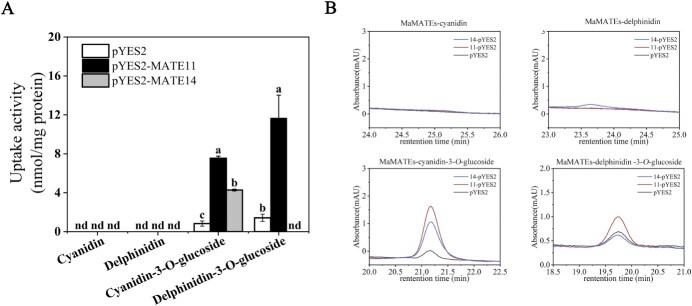
MaMATE11 and MaMATE14 transport cyanidin, delphinidin, cyanidin-3-*O*-glucoside, and delphinidin-3-*O*-glucoside in yeast microsomal vesicles. (A) Uptake of cyanidin, delphinidin, cyanidin-3-*O*-glucoside, and delphinidin-3-*O*-glucoside by yeast microsomal vesicles expressing MaMATE11 and MaMATE14. The uptake activities were calculated by subtracting the values obtained with and without MgATP. The data are expressed as means ± SD (*n* = 3), and distinct letters on the bars denote statistically significant differences (*P* <0.05, Tukey’s HSD test). (B) High Performance Liquid Chromatography (HPLC) analysis of cyanidin, delphinidin, cyanidin-3-*O*-glucoside, and delphinidin-3-*O*-glucoside taken up into microsomal vesicles isolated from yeast cells expressing MaMATE11 and MaMATE14.

Previous studies showed that *Arabidopsis* TT12 and *M. truncatula* MtMATE2 transport anthocyanin glucosides (cyanidin-3-*O*-glucoside) in yeast vesicles [7, 18, 21]. To determine whether MaMATE11 and MaMATE14 are anthocyanin transporters with similar transport activity *in vitro*, their full-length cDNAs were cloned into the pYES2 vector to create recombinant plasmids pYES2-MaMATE11 and pYES2-MaMATE14 and then expressed in yeast. Control experiments were performed using yeast cells carrying the empty pYES2 vector. Microsomal membrane vesicles were purified from the transformed yeast strains, and their membrane integrity was assessed through 9-amino-6-chloro-2-methoxyacridin (ACMA) fluorescence quenching assays [4, 6]. The result showed that the vesicles were present and intact and did not influence the results of further transport experiments (Fig. S6). Next, we conducted the initial transport studies with cyanidin, delphinidin, cyanidin-3-*O*-glucoside, and delphinidin-3-*O*-glucoside for the substrates. As illustrated in Fig. 3A and 3B, like the empty vector cells, MaMATE11- and MaMATE14-expressing membrane vesicles did not exhibit appreciable transport activity toward the anthocyanidins, cyanidin and delphinidin. Yeast cells expressing MaMATE11 took up both cyanidin-3-*O*-glucoside and delphinidin-3-*O*-glucoside, whereas yeast cells expressing MaMATE14 exclusively took up cyanidin-3-*O*-glucoside. Meanwhile, MaMATE11 had stronger transport activity than MaMATE14 with cyanidin-3-*O*-glucoside for the substrate. All of these transport experiments depended on MgATP as an energy source. Furthermore, parallel transport assays were conducted with individual supplementation of either vanadate (an ATPase inhibitor known to preferentially block ABC transporters) or NH_4_Cl (a vacuolar pH gradient disruptor). The result showed a strong decrease of NH_4_Cl in the uptake of cyanidin-3-*O*-glucoside by MaMATE11 or MaMATE14 and delphinidin-3-*O*-glucoside by MaMATE11, while there was no effect of vanadate (Table S3). Overall, these investigations showed MaMATE11 and MaMATE14 are ATP-dependent and function as vacuolar H^+^-dependent transporters *in vitro* to mediate anthocyanin glycoside transport but not anthocyanidins. However, their transport efficiency and substrate preferences may vary.

### Substrate specificity and transport efficiency of MaMATE11 and MaMATE14 in flavonoid transport

**Figure 4:**
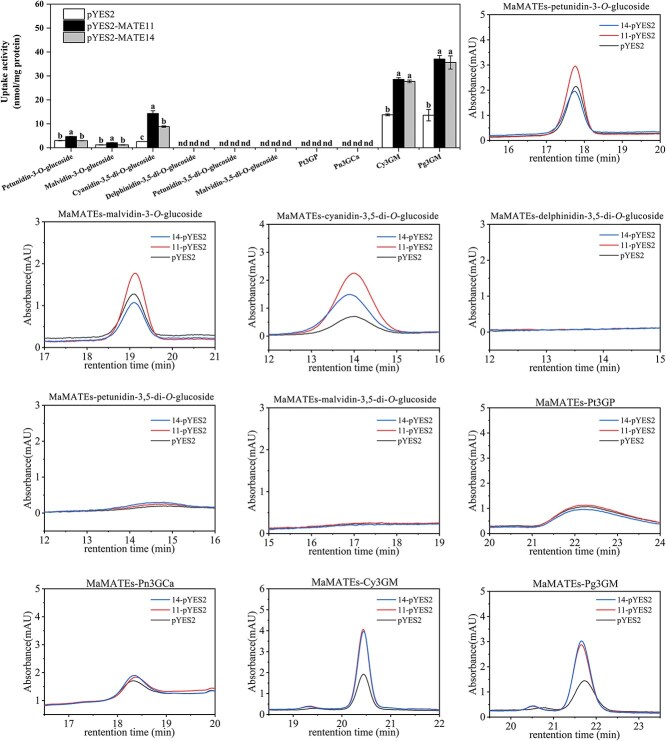
MaMATE11 and MaMATE14 transport other specifically glucosylated and acylated anthocyanins. (A) Uptake of petunidin-3-*O*-glucoside, malvidin-3-*O*-glucoside, cyanidin-3,5-di-*O*-glucoside, delphinidin-3,5-di-*O*-glucoside, petunidin-3,5-di-*O*-glucoside, malvidin-3,5-di-*O*-glucoside, Pt3GP, Pn3GCa, Cy3GM, and Pg3GM by yeast microsomal vesicles expressing MaMATE11 and MaMATE14. The uptake activities were calculated by subtracting the values obtained with and without MgATP. Results are presented as mean values ± SD of three samples with three replicates; different letters above the bars indicate significantly different values calculated by Tukey’s HSD tests (*P* <0.05). (B) HPLC analysis of petunidin-3-*O*-glucoside, malvidin-3-*O*-glucoside, cyanidin-3,5-di-*O*-glucoside, delphinidin-3,5-di-*O*-glucoside, petunidin-3,5-di-*O*-glucoside, malvidin-3,5-di-*O*-glucoside, Pt3GP, Pn3GCa, Cy3GM, and Pg3GM taken up into microsomal vesicles isolated from yeast cells expressing MaMATE11 and MaMATE14.

In this study, the anthocyanin components accumulated in ‘Dark Eyes’ (at Stage 4) were analyzed using UPLC-ESI-MS/MS by Metware Biotechnology Co., Ltd (Wuhan, China). The result revealed that, in addition to monoglucosyl anthocyanins, petunidin-3-*O*-glucoside, malvidin-3-*O*-glucoside, and delphinidin-3-*O*-glucoside, which mainly accumulated in blue flower grape hyacinth as previously reported [32], other diglucosyl, malonylated, and aromatic-acylated anthocynins were also detected, but to a lesser extent (Table S4). To verify the substrate specificity of MaMATE11 and MaMATE14, 10 main anthocyanin components identified in opening flowers of ‘Dark Eyes’ were used as substrates for further transport tests. The selected substrates were petunidin-3-*O*-glucoside, malvidin-3-*O*-glucoside, cyanidin-3,5-di-*O*-glucoside, delphinidin-3,5-di-*O*-glucoside, petunidin-3,5-di-*O*-glucoside, malvidin-3,5-di-*O*-glucoside, petunidin 3-(*p*-coumaroylrutinoside)-5-glucoside (Pt3GP), peonidin-3-*O*-(6-*O*-trans-caffeyl-2-*O*-β-glucopyranosyl-β-glucopyranoside)-5-*O*-β-glucopyranoside (Pn3GCa), cyanidin 3-*O*-(6-*O*-malonyl-β-D-glucoside) (Cy3GM), and pelargonidin 3-*O*-(6-*O*-malonyl-D-glucoside) (Pg3GM). HPLC profile of the standards is shown in Fig. S7. First, for anthocyanin monoglucosides, MaMATE11 transported the 3-*O*-glucosides of petunidin (petunidin-3-*O*-glucoside) and malvidin (malvidin-3-*O*-glucoside), but MaMATE14 did not take them up (Fig. 4). This is comparable to delphinidin-3-*O*-glucoside. These two MATE proteins transported cyanidin-3, 5-di-*O*-glucoside, an anthocyanin diglucoside, with MaMATE11 having a greater transport activity than MaMATE14. Using delphinidin-3, 5-di-*O*-glucoside, petunidin-3, 5-di-*O*-glucoside, and malvidin-3, 5-di-*O*-glucoside as the substrates did not result in any uptakes (Fig. 4). Notably, cyanidin 3-*O*-(6-*O*-malonyl-β-D-glucoside) and pelargonidin 3-*O*-(6-*O*-malonyl-D-glucoside) were taken up by both MaMATE11 and MaMATE14, and they demonstrated an absolute advantage in transport activity with these two malonylated anthocyanins for the substrates (Fig. 4). However, no transports were observed when two aromatic-acylated anthocyanins, petunidin 3-(*p*-coumaroylrutinoside)-5-glucoside and peonidin-3-*O*-(6-*O*-trans-caffeyl-2-*O*-β-glucopyranosyl-β-glucopyranoside)-5-*O*-β-glucopyranoside, were used as the substrates (Fig. 4). As described above, MaMATE11 and MaMATE14 had the substrate preferences of malonylated anthocyanins, while MaMATE11 might have the broader substrate spectrum and higher substrate affinities of anthocyanin glucosides than MaMATE14.

**Figure 5:**
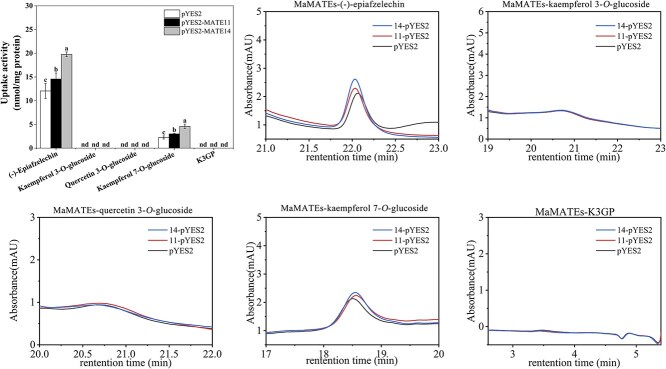
MaMATE11 and MaMATE14 transport other non-anthocyanin-type flavonoids. (A) Uptake of (−)-epiafzelechin, kaempferol 3-*O*-glucoside, quercetin 3-*O*-glucoside, kaempferol 7-*O*-glucoside, and K3GP by yeast microsomal vesicles expressing MaMATE11 and MaMATE14. The uptake activities were calculated by subtracting the values obtained with and without MgATP. The data are expressed as means ± SD (*n* = 3), and distinct letters on the bars denote statistically significant differences (*P* <0.05, Tukey’s HSD test). (B) The transport activity of MaMATE11 and MaMATE14 was evaluated by HPLC quantification of (−)-epiafzelechin, kaempferol 3-*O*-glucoside, quercetin 3-*O*-glucoside, kaempferol 7-*O*-glucoside, and K3GP in microsomal vesicles prepared from transformed yeast cells.

To investigate whether MaMATE11 and MaMATE14 could transport non-anthocyanin-type flavonoids, the transport experiments were performed with (−)-epiafzelechin, kaempferol 3-*O*-glucoside, quercetin 3-*O*-glucoside, kaempferol 7-*O*-glucoside, and kaempferol-3-*O*-(6″-*p*-coumaroyl) glucoside (K3GP) from the grape hyacinth blue flower as substrates. The results suggested that MaMATE11 and MaMATE14 could transport (−)-epiafzelechin and kaempferol 7-*O*-glucoside, but not kaempferol 3-*O*-glucoside, quercetin 3-*O*-glucoside, and kaempferol-3-*O*-(6″-*p*-coumaroyl) glucoside (Fig. 5). Furthermore, MaMATE14 had stronger transport activity for (−)-epiafzelechin and kaempferol 7-*O*-glucoside than MaMATE11, which has higher transport activity for anthocyanin glucosides.

Furthermore, cyanidin-3-*O*-glucoside, cyanidin-3, 5-di-*O*-glucoside, (−)-epiafzelechin, and kaempferol 7-*O*-glucoside were chosen for the transport kinetics analysis in order to confirm the variations in transport efficiency between MaMATE11 and MaMATE14. For the absorption of cyanidin-3-*O*-glucoside, MaMATE11 exhibited a *K*m of 57.46 μM and a *V*max of 0.66 nmol/min/mg protein, while MaMATE14 showed equivalent values of 61.40 μM and 0.54 nmol/min/mg protein for MaMATE14 (Fig. S8A and B). Uptake of cyanidin-3, 5-di-*O*-glucoside into vesicles from MaMATE11 was characterized by a *K*m of 26.98 μM and a *V*max of 0.73 nmol/min/mg protein, and from MaMATE14 it gave a *K*m of 45.32 μM and a *V*max of 0.48 nmol/min/mg protein (Fig. S8C and D).

Moreover, MaMATE11 and MaMATE14 also mediated the uptake of other flavonoids (Fig. S9), such as (−)-epiafzelechin (by MaMATE11, *K*m of 57.43 μM and *V*max of 1.25 nmol/min/mg protein; by MaMATE14, *K*m of 43.80 μM and *V*max of 1.46 nmol/min/mg protein) and kaempferol 7-*O*-glucoside (by MaMATE11, *K*m of 60.18 μM and *V*max of 0.34 nmol/min/mg protein; by MaMATE14, *K*m of 57.38 μM and *V*max of 0.38 nmol/min/mg protein). These results revealed that MaMATE11 had a higher transport affinity and efficiency than MaMATE14 with anthocyanin glucosides (cyanidin-3-*O*-glucoside and cyanidin-3, 5-di-*O*-glucoside). Instead, MaMATE14 demonstrated greater substrate selectivity for other flavonoids than MaMATE11. Overall, these data show that, while MaMATE11 and MaMATE14 have at least partially redundant transport actions on vacuolar flavonoid sequestration, they play distinct roles according to their substrate specificity and transport kinetics.

### Heterologous expression of *MaMATE11* and *MaMATE14* in *Arabidopsis* mutant

**Figure 6:**
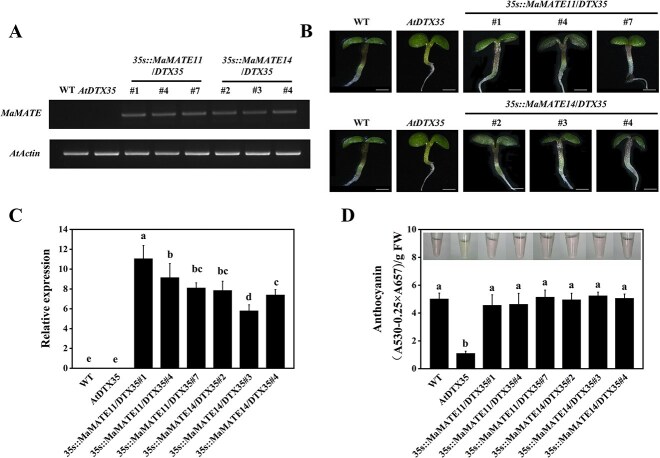
Functional complementation of *AtDTX35* mutant with *MaMATE11* and *MaMATE14*. Identification of the *Arabidopsis* transgenic lines overexpressing *MaMATE11* (Line 1, 4, and 7) and *MaMATE14* (Line 2, 3, and 4) by (A) RT-PCR and (C) qRT-PCR analysis. *AtActin* was the reference gene for normalizing the expression of these genes. (B) Phenotypic characterization and (D) Anthocyanin content of 7-day-old seedlings of *Arabidopsis* WT, *AtDTX35*, and transgenic lines. The data are expressed as means ± SD (*n* = 3), and distinct letters on the bars denote statistically significant differences (*P* <0.05, Tukey’s HSD test).

For functional validation of *MaMATE11* and *MaMATE14 in planta*, we used a complementation technique with the *Arabidopsis AtDTX35* mutant line, which is deficient in flavonoid transport [37]. Three independent transgenic lines constitutively overexpressing *MaMATE11* (Lines 1, 4, and 7) and *MaMATE14* (Lines 2, 3, and 4) were obtained and assessed for further investigation, respectively (Fig. 6A and C). Observing the phenotypic characteristics of the 7-day-old *Arabidopsis* seedlings revealed that the hypocotyls of *AtDTX35* seedlings were green, whereas those of the transgenic lines recovered purple pigmentation just like wild type (WT) (Fig. 6B). The anthocyanin content of WT, *AtDTX35*, and transgenic lines corresponded to their phenotypes (Fig. 6D). These results indicate that MaMATE11 and MaMATE14 could rescue the anthocyanin-deficient phenotype of the *AtDTX35* mutant and might be responsible for anthocyanin transport in grape hyacinth.

### Silencing of *MaMATE11* and *MaMATE14* block anthocyanin accumulation in grape hyacinth blue-colored flower

**Figure 7:**
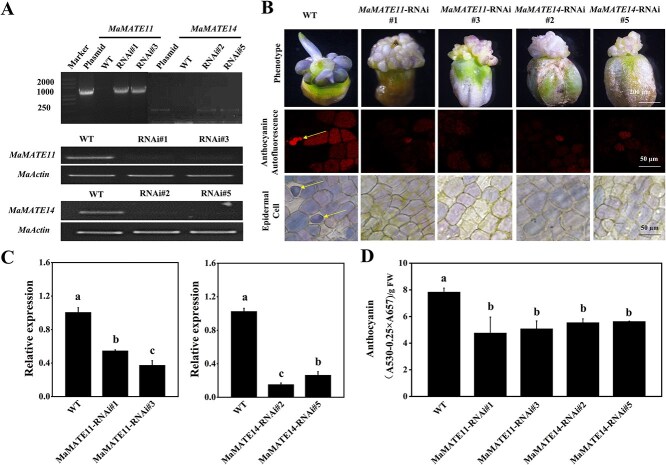
Silencing of the *MaMATE11* and *MaMATE14* genes in petals of blue-colored grape hyacinth. Identification of the RNAi transgenic lines of *MaMATE11* (Line 1, 3) and *MaMATE14* (Line 2, 5) by (A) PCR, RT-qPCR, and (C) qRT-PCR analysis. *MaActin* was the reference gene for normalizing the expression of these genes. (B) The phenotypes, the images of anthocyanins autofluorescence, and adaxial petal epidermal cells of fresh regenerative flower buds in WT, *MaMATE11* RNAi-silenced lines, and *MaMATE14* RNAi-silenced lines. AVIs are marked by yellow arrows. (D) Quantification of total anthocyanins in WT, *MaMATE11-*silenced lines, and *MaMATE14-*silenced lines. The data are expressed as means ± SD (*n* = 3), and distinct letters on the bars denote statistically significant differences (*P* <0.05, Tukey’s HSD test).

To further investigate and validate the functions of *MaMATE11* and *MaMATE14 in vivo*, we used an *Agrobacterium*-mediated transformation method based on the petal regeneration system of grape hyacinth [38]. *Agrobacterium tumefaciens* cultures containing *MaMATE11* or *MaMATE14* RNAi vectors were stably integrated into petals of *Muscari armeniacum*. Three months later, two RNAi transgenic lines of *MaMATE11* (Lines 1, 3) and *MaMATE14* (Lines 2, 5) were respectively identified using PCR amplification, RT-PCR (Fig. 7A), and qRT-PCR analysis (Fig. 7C). Compared to the nontransgenic controls, all transgenic petals had lower expression of *MaMATE11* and *MaMATE14*, resulting in a lighter violet color via inhibiting anthocyanin accumulation (Fig. 7B and D). In addition, lightmicroscopy and laser scanning confocal microscopy were used to investigate anthocyanin accumulation of the petals of controls and RNAi transgenic lines at the cellular levels. It should be noted that intensely colored intravacuolar bodies, anthocyanic vacuolar inclusions (AVIs), were observed in the cells of highly colored petals of the control (Fig. 7B). In contrast, only entirely soluble anthocyanins were seen in the cells of lighter violet-colored petals of *MaMATE11* or *MaMATE14* RNAi transgenic lines (Fig. 7B). The anthocyanin contents in the petals of RNAi transgenic lines were lower than those of the control (Fig. 7D). These findings revealed that MaMATE11 and MaMATE14 play essential roles in anthocyanin accumulation in grape hyacinth blue flowers.

### Activation of *MaMATE11* and *MaMATE14* by the anthocyanin regulators MaMybA and MaAN2

**Figure 8:**
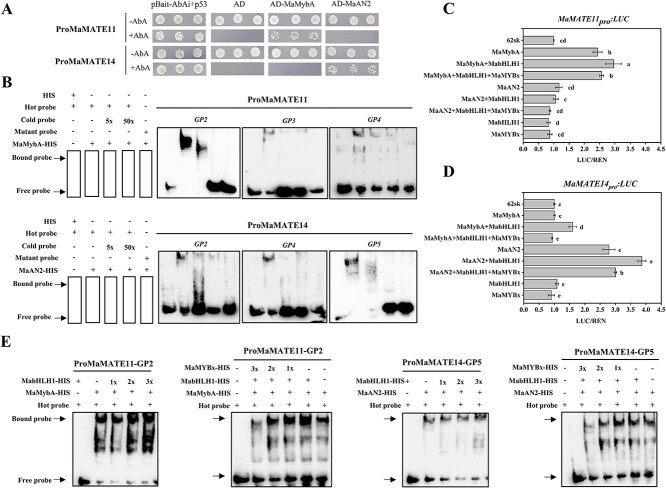
MaMybA/MaAN2 can respectively bind to *MaMATE11* and *MaMATE14* promoters to activate their expressions. (A) Y1H analysis confirmed the direct interaction of MaMybA/MaAN2 with *MaMATE11* and *MaMATE14* promoter sequences, with appropriate positive (pAbAi-p53 + pGADT7-p53) and negative (empty pGADT7) controls included in the experiment. (B) EMSA showing MaMybA could bind to CAACAA(GP2), GGATA (GP3), and CAACCA (GP4) elements of *ProMaMATE11*, while MaAN2 could bind to CAACCA (GP2), CAACAA (GP4), and CAACAG (GP5) elements of *ProMaMATE14.* ‘+’ indicates addition of the corresponding fusion protein or probe. ‘−’ indicates the lack of the corresponding fusion protein or probe. (C) Dual-luciferase assay showing MaMybA activated the promoters of *MaMATE11*. (D) Dual-luciferase assay showing MaAN2 activated the promoters of *MaMATE14*. MabHLH1 enhanced the transcriptional activation mediated by MaMybA and MaAN2, whereas MaMYBx attenuated the transcriptional activation of the MaMybA/MaAN2-MabHLH1 complex. The activation activity was evaluated through a LUC/REN ratio. Results are presented as mean values ± SD of three samples with three replicates; different letters above the bars indicate significantly different values calculated by Tukey’s HSD tests (*P* <0.05). (E) EMSA showing the effects of MabHLH1 and MaMYBx on the ability of MaMybA/MaAN2 to bind the fragments of *ProMaMATE11* (GP2) and *ProMaMATE14* (GP5), respectively. ‘1×’, ‘2×’, and ‘3×’ indicate the gradient addition of MabHLH1-HIS or MaMYBx-HIS.

Our earlier studies demonstrated that R2R3 MYB transcription factors, MaMybA and MaAN2 directly activated the expression of anthocyanin biosynthetic genes *MaDFR* and *MaANS*, indicating they are the primary anthocyanin regulatory genes in grape hyacinth [33, 34]. To determine whether MaMybA and MaAN2 bind to the promoters of *MaMATE11* and *MaMATE14 in vitro*, a yeast one-hybrid (Y1H) assay was performed. The results showed that MaMybA could directly bind to *ProMaMATE11* but not *ProMaMATE14*, whereas MaAN2 physically bound to *ProMaMATE14* but not *ProMaMATE11* (Fig. 8A).

To investigate the MYB-binding *cis*-elements, the promoter sequences of *MaMATE11* and *MaMATE14* were analyzed. As shown in Fig. S10, *ProMaMATE11* and *ProMaMATE14* contain predicted MYB-binding (MBS) elements: the MYB core element (CNGTTR) [39], Myb (NAACNN), the AC-rich element ([A/C]CC[A/T]A[A/C]) [34], or the MYBST1 element (GGATA) [40]. In addition, several light-responsive elements, auxin response elements, low-temperature response elements, anaerobic induction elements, etc. were present (Tables S5 and S6). To further verify the *cis*-elements or promoter regions that bind R2R3 MYB to *ProMaMATE11* and *ProMaMATE14*, the full-length promoters of *MaMATE11* and *MaMATE14* were divided into five fragments (GP1–GP5) according to the MBS elements (Fig. S10). The interactions between each fragment and corresponding transcription factor were measured by Y1H assays. The results showed that MaMybA could attach to the fragments GP2, GP3, and GP4 of *ProMaMATE11*, while MaAN2 bound to the fragments GP2, GP4, and GP5 of *ProMaMATE14* (Fig. S10). Electrophoretic mobility shift assay (EMSA) later confirmed the results. It revealed that MaMybA could bind to CAACAA (GP2), GGATA (GP3), and CAACCA (GP4) elements of *ProMaMATE11*, whereas MaAN2 could bind to CAACCA (GP2), CAACAA (GP4), and CAACAG (GP5) elements of *ProMaMATE14* (Fig. 8B). To verify whether the binding of MaMybA/MaAN2 could induce the transcriptional activities of promoters of *MaMATE11* or *MaMATE14 in vivo*, dual luciferase assays were performed in tobacco leaves. As shown in Fig. 8C and D, MaMybA demonstrated notable trans-activation effects on *ProMaMATE11* but not on *ProMaMATE14*. MaAN2 could activate *ProMaMATE14* but not *ProMaMATE11*. In the presence of MabHLH1, the trans-activation effects were stronger than infiltration of MaMybA or MaAN2 only. But when coinfiltrated with MaMYBx [26], the activation of *ProMaMATE11* or *ProMaMATE14* was repressed. These results were validated by the fluorescence image of promoter–luciferase assays in tobacco leaves (Fig. S11). Furthermore, EMSA experiments provided the validation of these results. As demonstrated in Fig. 8E, the binding affinity of the MaMybA/MaAN2 for the promoters of *MaMATE11* and *MaMATE14* exhibited a concentration-dependent increase upon addition of MabHLH1, but MaMYBx attenuated the binding activity of MaMybA/MaAN2-MabHLH1 to the MaMATE11 and MaMATE14 promoters, respectively. Taken together, we concluded that MaMybA and MaAN2, along with their cofactors MabHLH1 and MaMYBx, can synergistically regulate the expressions of *MaMATE11* and *MaMATE14*, respectively.

## Discussion

### Blue flowers have more complex anthocyanin transport pathways

The predominant anthocyanins in many naturally blue flowers, including cineraria (*Senecio cruentus*), delphinium (*Delphinium grandiflorum*), and campanula (*Campanula medium*), are modified with multiple aromatic acyl groups, a phenomenon termed polyacylation [31]. Polyacylated anthocyanins are thought to contribute to the production of a stable blue color in these flowers through intramolecular sandwich-type stacking of aromatic acyl residues onto the anthocyanidin chromophore [30]. By contrast, the main anthocyanins in blue *Muscari* flowers are simple delphinidin-type anthocyanins with one or two methyl groups [32], although small amounts of anthocyanins with other modifications (e.g. diglycosylation, malonylation, or aromatic-acylation) were also found. Anthocyanin biosynthesis occurs in the cytoplasm [41]; subsequent glycosylation and acylation modifications are crucial for color stabilization and may also serve as vacuolar targeting signals. These modifications may occur in the cytoplasm or the vacuole. Glycosylation is typically catalyzed by UDP-sugar-dependent GT family 1 anthocyanin glucosyltransferases (UAGTs) in the cytoplasm and by acylglucose-dependent glucosyltransferases of glycoside hydrolase family 1 (GH1-GTs) in the vacuole [42, 43]. Similarly, acylation is thought to be catalyzed by acyl-CoA-dependent BAHD acyltransferases (BAHD-ATs) in the cytoplasm but by serine carboxypeptidase-like acylglucose-dependent acyltransferases (SCPL-ATs) in the vacuole [43]. Modification of vacuolar anthocyanins by addition of an aromatic acyl-glucose concatemer chain has frequently been reported in blue flowers, catalyzed by GH1-GTs and SCPL-ATs [31], and we recently identified one SCPL-AT and two GH1-GTs involved in vacuolar anthocyanin modifications in blue *Muscari* [44, 45]. The relationship between anthocyanin modifications and anthocyanin transport remains to be clarified. Nonetheless, we speculate that blue flowers may have more complex anthocyanin transport pathways, because they must transport not only cytoplasmic anthocyanins but also precursor anthocyanins and acyl-glucose for vacuolar modification from the ER surface to the vacuole.

### MaMATE11 and MaMATE14 show partial functional redundancy but differ in substrate specificity and transport kinetics

Flavonoid transport is a coordinated, multistep process whose participating proteins show substantial functional redundancy. For example, the ABCC transporters ZmMRP4 and ZmMRP3 synergistically mediate anthocyanin sequestration in maize [16]; the MATE transporters AM1 and AM3 specifically transport acylated anthocyanins and have partially overlapping functions in grapevine [6]; and the homoeologous MATE transporters NtMATE21 and NtMATE22 modulate plant growth and flavonol transport in tobacco [23]. In this study, we investigated the roles of two MATE proteins in the vacuolar sequestration of flavonoids. The similar gene expression profiles (Fig. 1D) and 52.31% sequence identity (Fig. 1E) between MaMATE11 and MaMATE14 strongly suggested that they might exhibit at least partial functional redundancy. *In vitro* transport assays in yeast vesicles, complementation tests in the *Arabidopsis AtDTX35* mutant, and gene silencing in *Muscari* flower petals all confirmed that MaMATE11 and MaMATE14 have partially overlapping functions in flavonoid transport.

Several lines of evidence suggest that different members of a single transporter family can differ in flavonoid substrate specificity and transport kinetics. In *M. truncatula*, MATE1 transported epicatechin 3′-*O*-glucoside at a high efficiency and cyanidin-3-*O*-glucoside at a lower efficiency [21], whereas MATE2 transported anthocyanin and flavone glucosides, particularly malonylated flavonoid glucosides [18]. In the present study, *in vitro* transport assays suggested that MaMATE11 had a broader substrate spectrum and higher substrate affinities for anthocyanin glucosides than MaMATE14, despite the fact that both proteins preferentially transported malonylated anthocyanins (Figs 3 and 4; Fig. S8). In previous work, we found that blue grape hyacinth flowers contained mainly petunidin-3-*O*-glucoside, malvidin-3-*O*-glucoside, and delphinidin-3-*O*-glucoside [32]. The relative contents of these anthocyanins gradually increased during flower development, peaking at Stage 4 (54%, 16%, and 10%, respectively) and slightly decreasing at Stage 5. In the present study, *MaMATE11* expression and anthocyanin content showed similar changes over the course of flower development in ‘Dark Eyes’ (Fig. 1D), suggesting that MaMATE11 might be involved in transport of anthocyanins into vacuoles. Transport experiments confirmed that MaMATE11 could transport petunidin-3-*O*-glucoside, malvidin-3-*O*-glucoside, and delphinidin-3-*O*-glucoside, whereas MaMATE14 could not (Figs 3 and 4). Thus, our results suggest that these two MATE transporters exhibit partially overlapping transport functions but have distinct substrate specificities and transport kinetics.

The substrate preferences of flavonoid transport proteins have long been a matter of debate. Recent studies indicate that transporter–substrate affinity depends largely on flavonoid side-chain chemistry. In grapevine, e.g. acylated anthocyanins are transported by the MATE transporters AM1 and AM3, whereas glucosylated anthocyanins are transported by the ATP-binding cassette protein ABCC1 [4, 6]. Here, we found that MaMATE11 and MaMATE14 could transport multiple glucosylated and acylated anthocyanins, as well as some non-anthocyanin flavonoids, *in vitro*. Both transporters showed a preference for malonylated anthocyanin substrates, similar to MATE2 from *M. truncatula* [18] and AM1/3 from grapevine [6], suggesting that MATE transporters might play a key role in malonylated anthocyanin transport. It should be noted that vesicles expressing the empty vector (pYES2) also showed prominent transport signals with some substrates, including malonylated anthocyanins (Fig. 4), perhaps because of nonspecific membrane–substrate interactions. Similar phenomena have been reported previously [6]. Whether homologs of MaMATE11/14 or ABCC transporters are involved in the transport of other flavonoids remains to be clarified, and the specific proteins that participate in vesicle-mediated flavonoid transport remain to be identified. Notably, MaMATE11 and MaMATE14 were not involved in the transport of aromatic-acylated anthocyanins (petunidin 3-(*p*-coumaroylrutinoside)-5-glucoside and peonidin-3-*O*-(6-*O*-trans-caffeyl-2-*O*-β-glucopyranosyl-β-glucopyranoside)-5-*O*-β-glucopyranoside; Fig. 4), suggesting that aromatic-acylated decoration of anthocyanins may take place in the vacuole after transmembrane transport. Because non-acylated, delphinidin-type anthocyanins are the predominant anthocyanins in blue grape hyacinth flowers (~80% at Stage 4) [32], we suspect that acylated anthocyanins accounted for <20% of total anthocyanins. Silencing of *MaMATE11* and *MaMATE14* by RNA interference reduced total anthocyanin accumulation in blue grape hyacinth flowers, likely by reducing the content of non-acylated anthocyanins, because the proportion of acylated anthocyanins is very low. Aromatic-acylated decoration of anthocyanins has been proposed to be associated with the formation of AVIs [46]. AVIs were not observed in petal cells of MaMATE11/14 RNAi transgenic lines (Fig. 7B), despite the fact that MaMATE11/14 do not appear to transport aromatic-acylated anthocyanins. We propose that this can be attributed mainly to the reduction in total anthocyanin content, because AVI formation requires not only the accumulation of aromatically acylated anthocyanins but also high levels of anthocyanin production in general [46].

A small amount of pigmentation was still observed in MaMATE11/14 RNAi transgenic lines. This may be due to (i) incomplete transcriptional suppression by RNAi-mediated knockdown, and/or (ii) functional redundancy, whereby knockdown of a single transporter gene may not fully block vacuolar anthocyanin sequestration. In future work, we will simultaneously knock out both *MaMATE11/14* genes, as well as other transporter genes (e.g. *GSTs*), to comprehensively evaluate their combined effects on pigment accumulation in grape hyacinth. In addition, a previous study reported that the glutathione S-transferase VvGSTU60 interacts and functionally cooperates with the MATE transporter VvDTX41B, significantly enhancing proanthocyanidin accumulation in grapes [47]. Future studies will aim to determine whether similar cooperative mechanisms exist between MaMATE11 and MaMATE14 and whether MATE–GST interactions also contribute to anthocyanin trafficking.

### Transcriptional regulation of flavonoid transporters

Numerous studies have shown that MBW complexes control the expression of flavonoid biosynthesis genes [25]. Different groups of MYBs specifically regulate distinct branches of flavonoid biosynthesis [39], and many *GST*, *ABCC*, and *MATE* transporter genes are also regulated by R2R3-MYB proteins. Nonetheless, the transcriptional regulation of genes encoding vacuolar flavonoid transporters remains to be fully characterized.

Studies spanning two decades have revealed a dual role for MYBs in the regulation of flavonoid biosynthesis and transport, with ANTHOCYANIN MUTANT controlling SlMTP77 in anthocyanin pathways [48] and NtMYB12 regulating the flavonol transporters NtMATE21/22 [23]. Here, we demonstrated that MaMybA and MaAN2, transcriptional activators of genes involved in anthocyanin biosynthesis, also bind to the promoters of *MaMATE11* and *MaMATE14*, respectively, regulating their transcriptional activity in conjunction with MabHLH1 and the R3-MYB repressor MaMYBx (Fig. 8). Our findings thus suggest that the transcription of vacuolar transporters is directly regulated by the same MYB transcription factors that target anthocyanin biosynthesis genes. Dual-luciferase and EMSA experiments provided evidence that an MaMybA/MaAN2–MabHLH1–MaMYBx transcriptional regulatory network controls expression of the flavonoid transporter genes *MaMATE11/14* (Fig. 8C–E). To the best of our knowledge, this is the first report of a complete transcriptional regulatory network for a flavonoid transport pathway, comprising both transcriptional activators and repressors.

**Figure 9:**
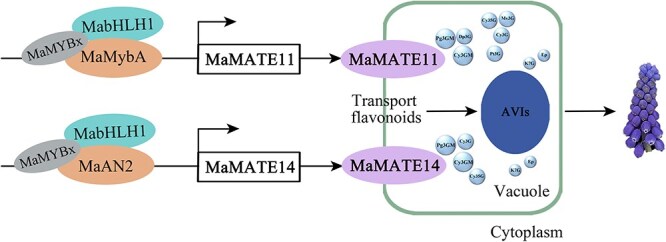
Suggested model of MaMATE11 and MaMATE14 mediate flavonoid transport in grape hyacinth blue-colored flower.

Overall, we propose a model in which MaMATE11/14 mediate the vacuolar transport of flavonoids in blue flowers of grape hyacinth (Fig. 9). MaMATE11 and MaMATE14 localize to the tonoplast in petal epidermal cells. *In vitro* transport experiments showed that MaMATE11 can transport cyanidin-3-*O*-glucoside, delphinidin-3-*O*-glucoside, petunidin-3-*O*-glucoside, malvidin-3-*O*-glucoside, cyanidin-3,5-di-*O*-glucoside, Cy3GM, Pg3GM, (−)-epiafzelechin, and kaempferol 7-*O*-glucoside across the tonoplast, whereas MaMATE14 can transport cyanidin-3-*O*-glucoside, cyanidin-3,5-di-*O*-glucoside, cyanidin 3-*O*-(6-*O*-malonyl-β-D-glucoside), pelargonidin 3-*O*-(6-*O*-malonyl-D-glucoside), (−)-epiafzelechin, and kaempferol 7-*O*-glucoside. Both transporters preferentially transport malonylated anthocyanins. Suppressing *MaMATE11* and *MaMATE14* expression altered petal color, anthocyanin accumulation, and the presence of AVIs in blue grape hyacinth flowers. Finally, we demonstrated that an MaMybA/MaAN2–MabHLH1–MaMYBx transcriptional network regulates the expression of *MaMATE11* and *MaMATE14*. MaMybA and MaAN2 bind to the promoters of *MaMATE11* and *MaMATE14*, respectively, activating their expression, and this activation is enhanced by the presence of MabHLH1 but repressed by MaMYBx.

## Materials and methods

### Plant materials

Grape hyacinth cultivars used in this study, including a blue-colored cultivar *Muscari aucheri* ‘Dark Eyes’ (MB) and a white-colored cultivar *M. aucheri* ‘White Magic’ (MW), are grown in a greenhouse of the Northwest A&F University in Yangling District, Shaanxi Province, China. Five floral developmental stages were determined in accordance with previous descriptions (Fig. 1C) [32]. Fresh buds or petals, roots, bulbs, and leaves were stored at −80°C until use. *Arabidopsis* Col-0 WT, AtDTX35 mutant, and transgenic *Arabidopsis* and *N. benthamiana* plants were cultivated in growth chambers at 22°C under a photoperiod of 16-h light/8-h dark.

### RNA-Seq analysis

To identify differentially expressed MATE genes, fresh petal samples at Stage 4 of floral development from 10 flower spikes of ‘Dark Eyes’ and ‘White Magic’ were randomly selected for each pool. To ensure data reliability, triplicate RNA-Seq assays were performed for each sample group. The RNA-Seq libraries were prepared from all specimens at Metware Biotechnology Co., Ltd. (Wuhan, China). The data was initially filtered to obtain clean data. Gene expression levels were quantified using FPKM normalization. DESeq2 analysis was performed to identify the differentially expressed genes (DEGs) between samples. The significant DEGs were screened out based on the log2FC values ≧ 1 and false discovery rate (FDR) < 0.05. A heatmap was plotted with log2FPKM values and normalized to visually illustrate the different expression levels. The raw data files are deposited in the NCBI Sequence Read Archive (SRA) database with an accession number PRJNA1162263.

### Bioinformatic analysis

Phylogenetic analysis using MATE transporter protein sequences from different plants. The phylogenetic tree was constructed using the MEGA 7.0 software with the neighbor-joining algorithm and 1000 bootstrap replicates. Sequence alignment was performed using GenDoc software. The protein transmembrane domains were predicted using the TMHMM 2.0 software (http://www.cbs.dtu.dk/services/TMHMM/). Detailed sequence accession information appears in Table S1.

### Gene cloning, RT-PCR, and qRT-PCR analysis

We followed the previously established protocol for RNA purification and cDNA synthesis [26]. The MaMATE11 and MaMATE14 genes were cloned from the cDNA of ‘Dark Eyes’ petals using PrimeSTAR HS DNA polymerase (TaKaRa, Dalian, China). qRT-PCR was conducted on a Bio-Rad IQ5 system (95°C 3 min; 40 cycles of 95°C 10 s/58°C 20 s), with melt curve verification and 2^−ΔΔCT^ analysis. *MaActin* and *AtActin* served as the internal control genes for grape hyacinth and *Arabidopsis* samples, respectively. The data showing the stable expression of *MaActin* under our experimental conditions is listed in Table S7. qRT-PCR analysis was performed using three samples, with three replicates. The primers used for gene cloning, RT-PCR, and qRT-PCR are listed in Table S2.

### Anthocyanin extraction and measurement

Approximately 0.2 g of tissues were ground to powder in liquid nitrogen and then added to 1 ml of extraction solution (methanol, ddH_2_O, formic acid, and trifluoroacetic acid, 70:27:2:1, v/v). All samples were extracted and measured as previously described, using a UV-Visible Spectrophotometer (UV2600; Shimadzu, Kyoto, Japan) [34]. The subtracted absorbance was calculated as A530 (peak absorption of anthocyanins) – 0.25 × A657 (maximum absorption of chlorophyll-degradation products). Each measurement was performed in triplicate to ensure reproducibility.

### Subcellular localization analysis

To determine the subcellular localization of MaMATE11/14, their coding sequences (excluding stop codons) were fused to GFP in the pCAMBIA2300-GFP vector (*35s*::GFP), creating the overexpression vectors *35s*::*MaMATE11*-GFP and *35s*::*MaMATE14*-GFP. The PCR primers used are listed in Table S2. *35s*::GFP, *35s*::*MaMATE11*-GFP and *35s*::*MaMATE14*-GFP, were introduced into *A. tumefaciens* (GV3101 strain) and coinfiltrated along with the γ-TIP tonoplast marker fused to the mCherry (a red fluorescent protein) into *N. benthamiana* leaves and purple onion bulb epidermis cells. Fluorescence signals for GFP and mCherry were analyzed by confocal laser-scanning microscopy (TCS SP8; Leica, Wetzlar, Germany) 16–48 h postinfiltration.

### Yeast vesicle isolation and transport activity measurements

The full-length cDNAs of *MaMATE11* and *MaMATE14* were cloned into the yeast expression vectors pYES2 via the *BamH*I and *EcoR*I sites, yielding pYES2-MaMATE11 and pYES2-MaMATE14, which were subsequently transformed into *Saccharomyces cerevisiae* strain BY4741. The primers are presented in Table S2. Yeast microsome isolation for transport assays followed established protocols [49]. The physiological tightness of the vesicle fractions was evaluated by fluorescence quenching of the dye ACMA [4].

Uptake experiments to study the transport of flavonoid substrates into yeast vesicles were performed as previously described [18]. The 1 ml reaction solution contained 25 mM Tris-MES (pH 8.0), 0.4 M sorbitol, 50 mM KCl, 5 mM MgATP, and 0.1% (w/v) BSA. At 25°C, the assays were initiated by the addition of membrane vesicles (protein content, 100 μg) and transport substrate (100 μM) into the reaction solution, followed by brief agitation with a vortex mixer. After 20 min, the reactions were terminated with 10 ml of ice-cold transport buffer, including 25 mM Tris-MES (pH 8.0) and 0.4 M sorbitol. The reaction mixtures were immediately loaded on the prewetted 0.22 μm PVDF membrane filters, and the filter-bound flavonoids were dissolved using 1 ml of 50% (v/v) methanol. The eluted flavonoids were then quantified by HPLC as previously published [33]. HPLC separation was performed on a Shimadzu LC-2030C liquid chromatograph with a diode array detector (Shimadzu, Kyoto, Japan) and an Inertsil C-18 column (5.0 μm particle size, 4.6 × 250 mm) (Shimadzu, Tokyo, Japan). The solvents utilized were A, 0.1% formic acid in water, and B, methanol. The oven temperature was set to 30°C, and the flow rate was 0.6 ml/min. The elution gradient was as follows: 0 min, 5% Solution B; 5 min, 20% Solution B; 10 min, 30% Solution B; 15 min, 40% Solution B; and 30 min, 5% Solution B. Quantitation was performed at 520 nm for anthocyanin, 280 nm for (−)-Epiafzelechin, and 254 nm for the rest of the flavonoids. MgATP was omitted from the non-energized controls. The uptake activities were calculated by subtracting the values obtained with and without MgATP. Inhibitor assays were conducted with 1 mM vanadate or 5 mM NH_4_Cl in the reaction mixture. All assays were performed using three separate vesicle isolations.

To determine the transport kinetics, transport assays were performed with different concentrations of cyanidin-3-*O*-glucoside, cyanidin-3, 5-di-*O*-glucoside, (−)-epiafzelechin, and kaempferol 7-*O*-glucoside (from 25 to 200 mM) in the presence or absence of 5 mM MgATP. After 20 min of incubation at 25°C, the transported flavonoids were measured by HPLC as described above. Lineweaver–Burk plots were used to calculate *Km* and *V*max values. The net speeds were calculated by subtracting the values obtained with and without MgATP.

In this study, (−)-Epiafzelechin was purchased from Macklin Biological Technology Co., Ltd. (Shanghai, China). The other anthocyanin and flavonol standards were obtained from Solarbio Science & Technology Co., Ltd. (Beijing, China).

### *Arabidopsis* complementation analysis

The *Arabidopsis* AtDTX35 mutant (SALK_095202C) was transformed with *35s::MaMATE11*-GFP and *35s::MaMATE14*-GFP constructs via the floral dip method [50]. Transgenic lines were selected on kanamycin-containing (50 mg/ml) one-half Murashige and Skoog (MS) medium. Untransformed WT *Arabidopsis* (Col-0) and *AtDTX35* were used as controls.

### Stable transformation of grape hyacinth

To create RNAi constructs, 300-bp specific fragments of *MaMATE11* and *MaMATE14* were inserted as inverted repeats into the pFGC5941 vector (*Nco*I/*Swa*I and *BamH*I/*Xba*I sites), creating pFGC-*MaMATE11* RNAi and pFGC-*MaMATE14* RNAi. These recombinant plasmids were introduced into *Agrobacterium* strain GV3101 and then transformed into grape hyacinth (*M. armeniacum*) flower buds via *Agrobacterium*-mediated methods [38]. After 3 months of selection, adaxial epidermal sections from regenerated flower buds of WT controls and RNAi transgenic lines were prepared and analyzed using a fluorescent microscope (Olympus BX63; Tokyo, Japan) and a confocal laser scanning microscope (TCS SP8; Leica, Wetzlar, Germany).

### Analysis of *cis*-regulatory elements in the promoters of *MaMATE11* and *MaMATE14*

The promoter regions of *MaMATE11* and *MaMATE14* were cloned from ‘Dark Eyes’ genomic DNA (gDNA) using the genome walker kit (Clontech, USA). The primers are listed in Table S2. The *cis*-regulatory elements were predicted on the PlantCARE database (http://bioinformatics.psb.ugent.be/webtools/plantcare/html/).

### Yeast one-hybrid assay

To study promoter interactions, 901- and 1025-bp promoter regions of *MaMATE11* (*ProMaMATE11*; OQ185276) and *MaMATE14* (*ProMaMATE14*; OQ185277), along with truncated variants (GP1–5), were cloned into the yeast vector pAbAi. Meanwhile, the *MaMybA* and *MaAN2* coding sequences were fused to the pGADT7 vector, resulting in AD-MaMybA and AD-MaAN2. The primers used are listed in Table S2. Linearized pAbAi constructs were transformed into Y1H Gold yeast, and the minimal inhibitory AbA concentrations were determined on SD/-Ura plates (Fig. S12). Subsequently, AD-MaMybA and AD-MaAN2 were introduced into bait-containing yeast and plated on SD/−Leu + AbA. The pAbAi-p53+ pGADT7-p53 was used as a positive control, whereas the empty pGADT7 vector served as a negative control.

### Electrophoretic mobility shift assay

The ORFs of *MaMybA*, *MaAN2*, and *MaMYBx* were cloned into the pCold TF vector, respectively. MabHLH1 coding sequences were inserted into the pET-32a vector. The recombinant protein expression and purification was performed as previously described [51]. DNA probes with 5′-biotin modifications were synthesized from Sangon Biotech (http://www.sangon.com/). Biotin-labeled DNA was detected using the Light Shift Chemiluminescence EMSA Kit (ThermoFisher Scientific, USA). The primers and EMSA probes used are presented in Table S2. The images were obtained using the chemiluminescence and spectral fluorescence imaging system (Alliance Q9 Advanced; UVItec, Cambridge, UK).

### Dual-luciferase assay

Dual-luciferase reporter assays were conducted as previously described [26]. The *MaMATE11* and *MaMATE14* promoters were fused to the pGreenII 0800-LUC vector, and the coding sequences for MaMybA, MaAN2, MaMYBx, and MabHLH1 were placed into the pGreenII 62-SK vector. The primers are listed in Table S2. These constructs were introduced into *Agrobacterium* GV3101 via freeze–thaw transformation and then infiltrated into 4–6 leaf-stage *N. benthamiana*. *Nicotiana benthamiana* leaves were sprayed with 1 mM D-luciferin and imaged after 5–10 min of dark adaptation using a PlantView100 imager (Biolight Biotechnology, Guangzhou, China). Quantitative LUC/REN ratios were determined with a Dual Luciferase Kit (Yeasen Biotechnology, Shanghai, China) on a Tecan Infinite M200 system (Männedorf, Switzerland), with triplicate biological measurements.

## Supplementary Material

Web_Material_uhaf270
