# Supplementary material for: The vacuolar transporters MaMATE11 and MaMATE14 affect blue flower coloration in grape hyacinth (Muscari)
Source: Hortic Res. 2025 Oct 17;13(1):uhaf270. doi: 10.1093/hr/uhaf270 (PMC12863215; doi:10.1093/hr/uhaf270)
Supplement: Web_Material_uhaf270 [file web_material_uhaf270.zip › supplementary figures S1-S12.docx]

# The vacuolar transporters MaMATE11 and MaMATE14 affect blue flower coloration in grape hyacinth (*Muscari*)

**Xiaoyun Cao^1,2,3,^**^†^**, Jingwen Xie^1,2,3,^**^†^**, Xuelan Gao^1,2,3^, Wanqi Pan^1,2,3^, Jiaxin Gong^1,2,3^, Lingjuan Du^1,2,3*^**

^1^ College of Landscape Architecture and Arts, Northwest A&F University, Yangling 712100, Shaanxi, PR China

^2^ State Key Laboratory of Crop Stress Resistance and High-Efficiency Production,

Northwest A&F University, Yangling 712100, Shaanxi, PR China

^3^ Key Laboratory of Horticultural Plant Biology and Germplasm Innovation in Northwest China, Ministry of Agriculture, Yangling 712100, Shaanxi, PR China

† These authors contributed equally to this study.

* Correspondence: dulingjuan@nwafu.edu.cn

# Supplementary data


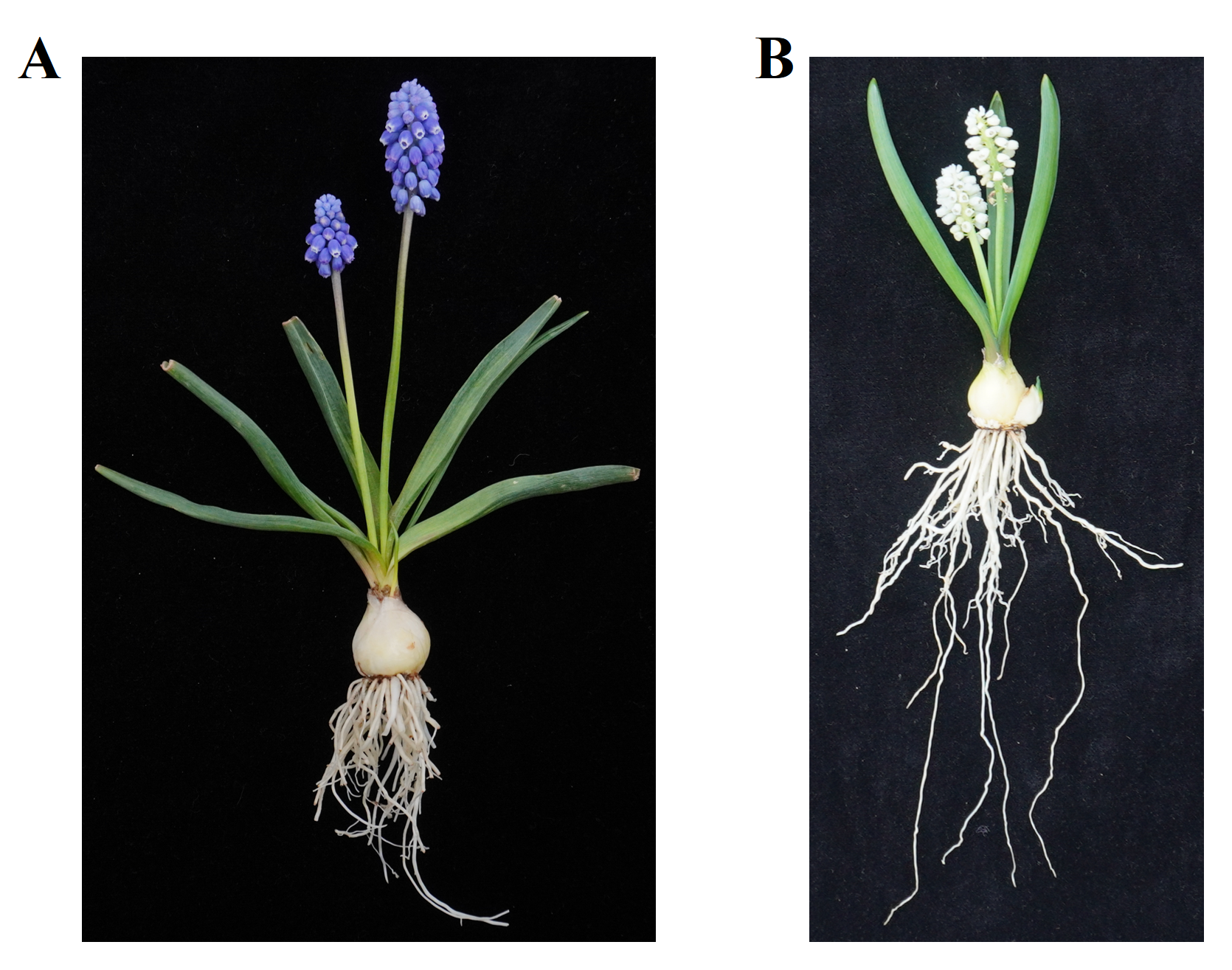


**Fig. S1.** Phenotypes of the blue-colored cultivar *M. aucheri* ‘Dark Eyes’ (A) and the white-colored cultivar *M. aucheri* ‘White Magic’ (B).

**
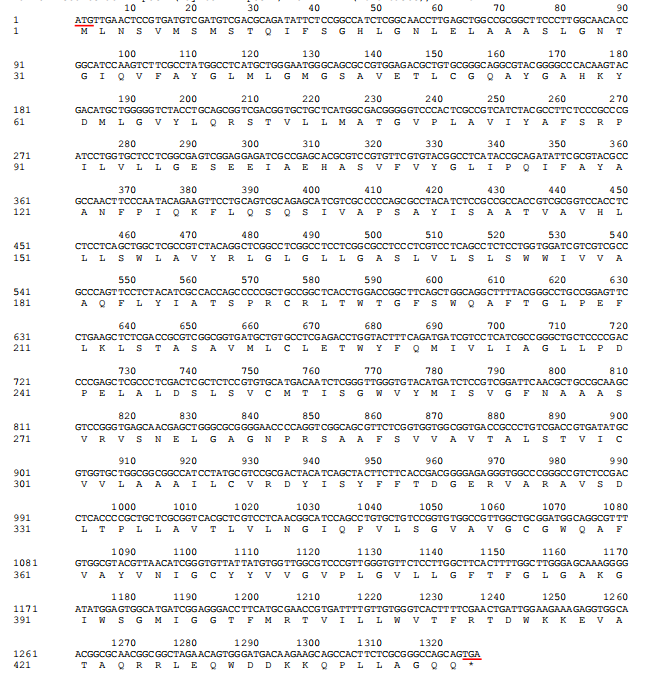
**

**Fig. S2.** The nucleotide and deduced amino acid sequences of MaMATE11.


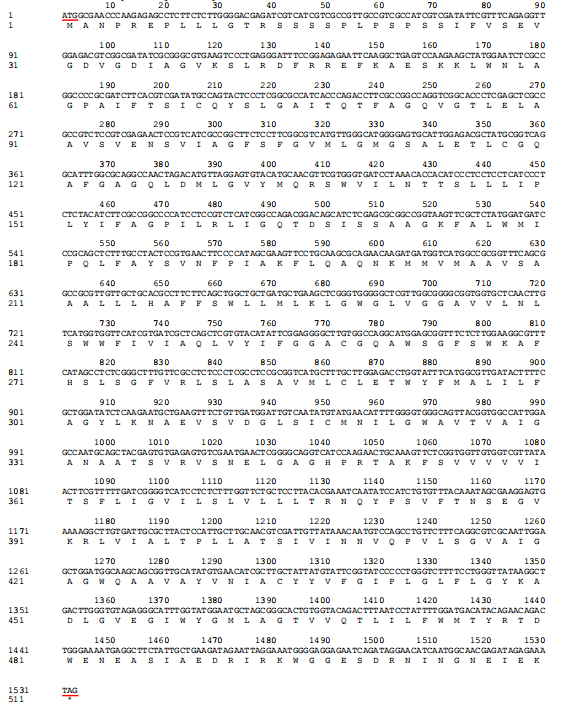


**Fig. S3.** The nucleotide and deduced amino acid sequences of MaMATE14.





**Fig. S4.** Transmembrane domains of MaMATE11 and MaMATE14 proteins.


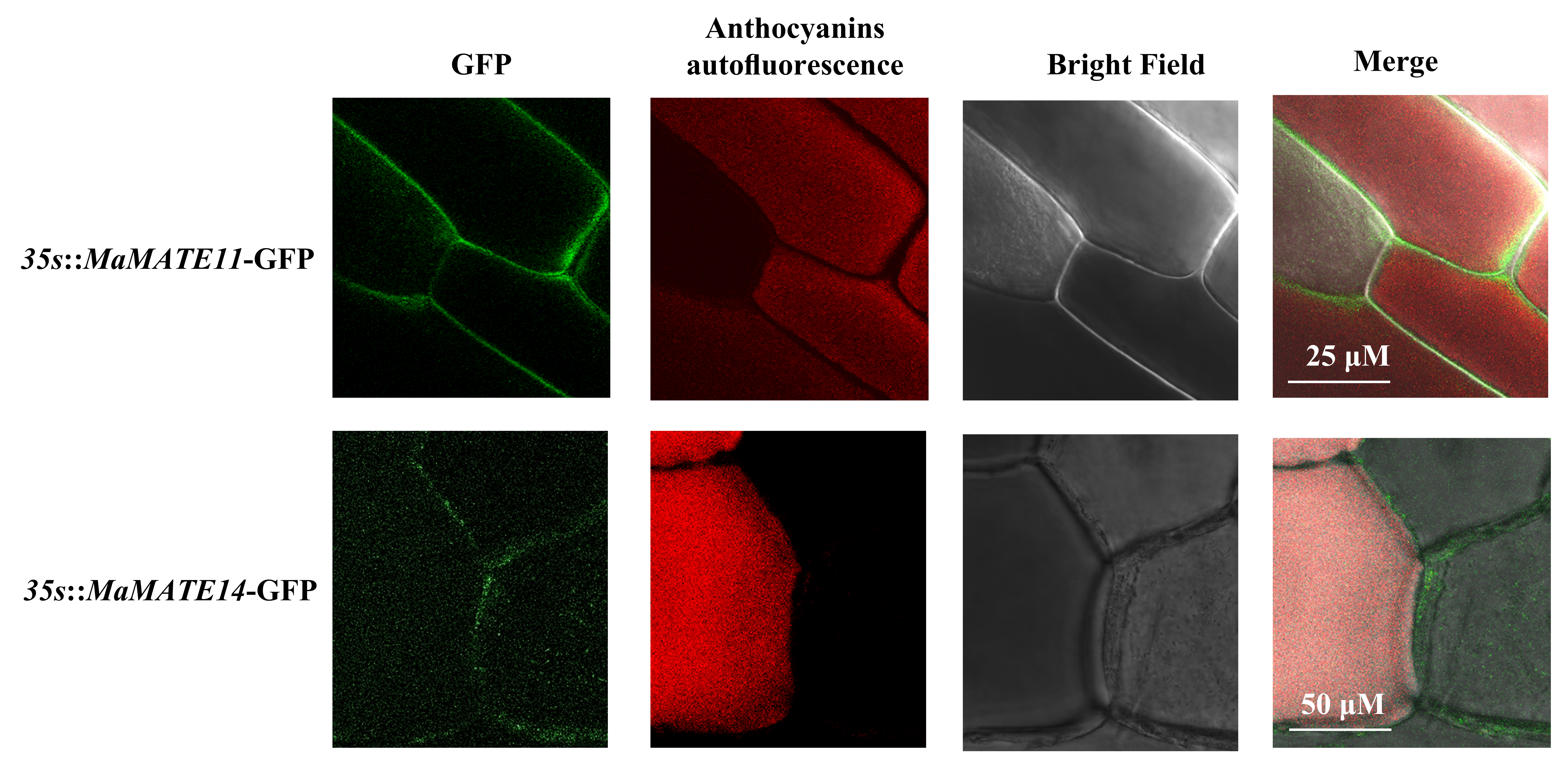


**Fig. S5.** Subcellular localization of MaMATE11 and MaMATE14 proteins in purple onion bulb epidermis cells (with anthocyanins).

**Fig. S6.** Assessment of physiological tightness of yeast microsomes by ATP-dependent ﬂuorescence quenching of the dye ACMA. It showed the vesicles of pYES2, pYES2-MaMATE11, and pYES2-MaMATE14 were present and intact, and the pH gradient is generated. The basic fluorescence was restored by addition of (NH4)_2_SO_4_, which indicated that the proton gradient formed across the membranes collapsed and the vesicles were intact. The physiological tightness was checked when prepared the microsomes each time.


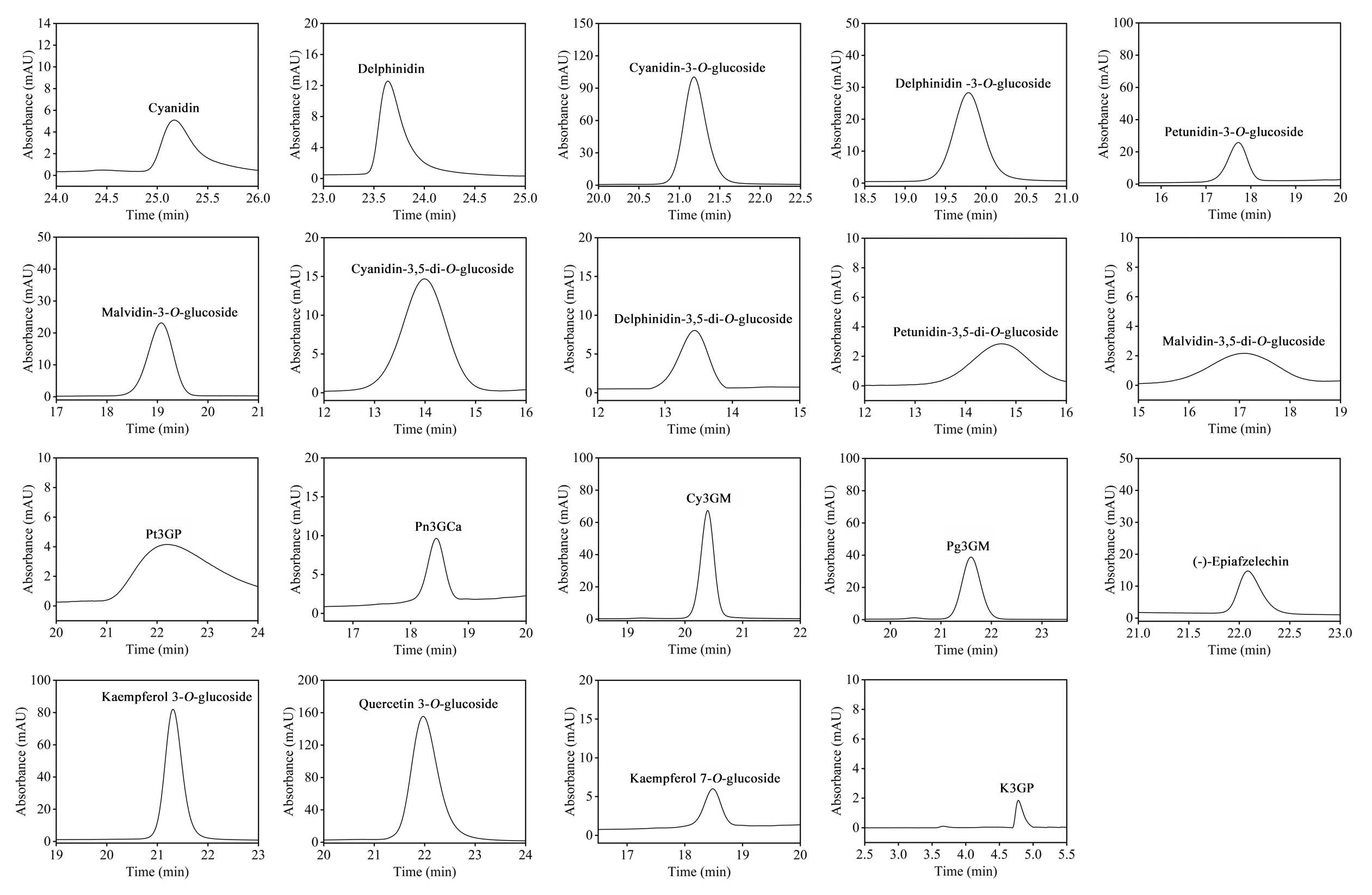


**Fig. S7.** HPLC profile of the standards.


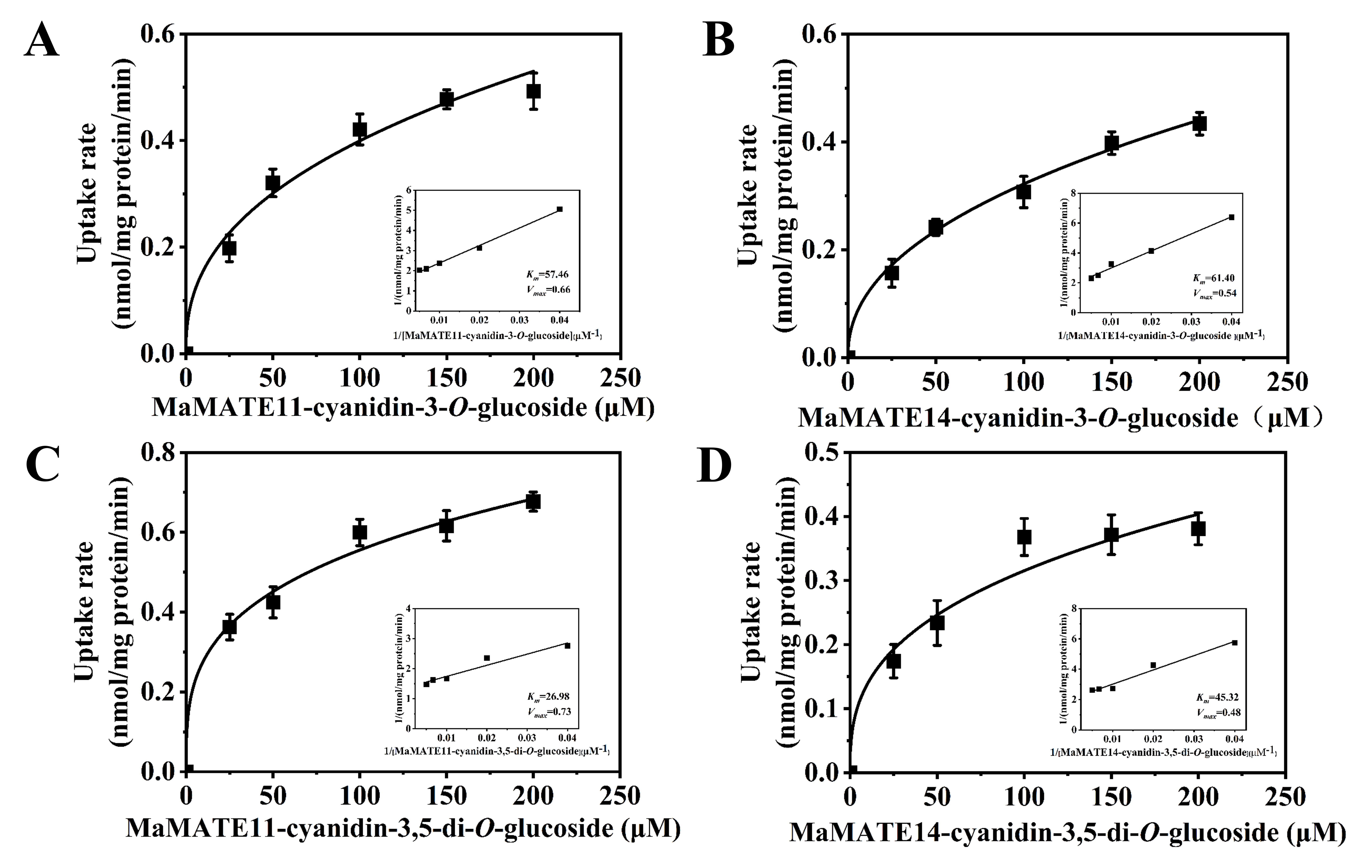


**Fig. S8.** Kinetics of uptake of cyanidin-3-*O*-glucoside and cyanidin-3, 5-di-*O*-glucoside by yeast microsomal vesicles expressing MaMATE11 and MaMATE14. (A) and (C) Concentration dependence of uptake of cyanidin-3-*O*-glucoside and cyanidin-3, 5-di-*O*-glucoside into vesicles from yeast expressing MaMATE11. Double reciprocal plots of initial rate data at different concentrations of cyanidin-3-*O*-glucoside and cyanidin-3, 5-di-*O*-glucoside by MaMATE11. (B) and (D) Concentration dependence of uptake of cyanidin-3-*O*-glucoside and cyanidin-3, 5-di-*O*-glucoside into vesicles from yeast expressing MaMATE14. Double reciprocal plots of initial rate data at different concentrations of cyanidin-3-*O*-glucoside and cyanidin-3, 5-di-*O*-glucoside by MaMATE14. Results are presented as net transport, calculated by subtracting the values measured in the absence of ATP from the values measured in the presence of ATP after 20 min. Data are mean ± standard deviation (SD) in three replicate uptake assays from three independent membrane preparations.


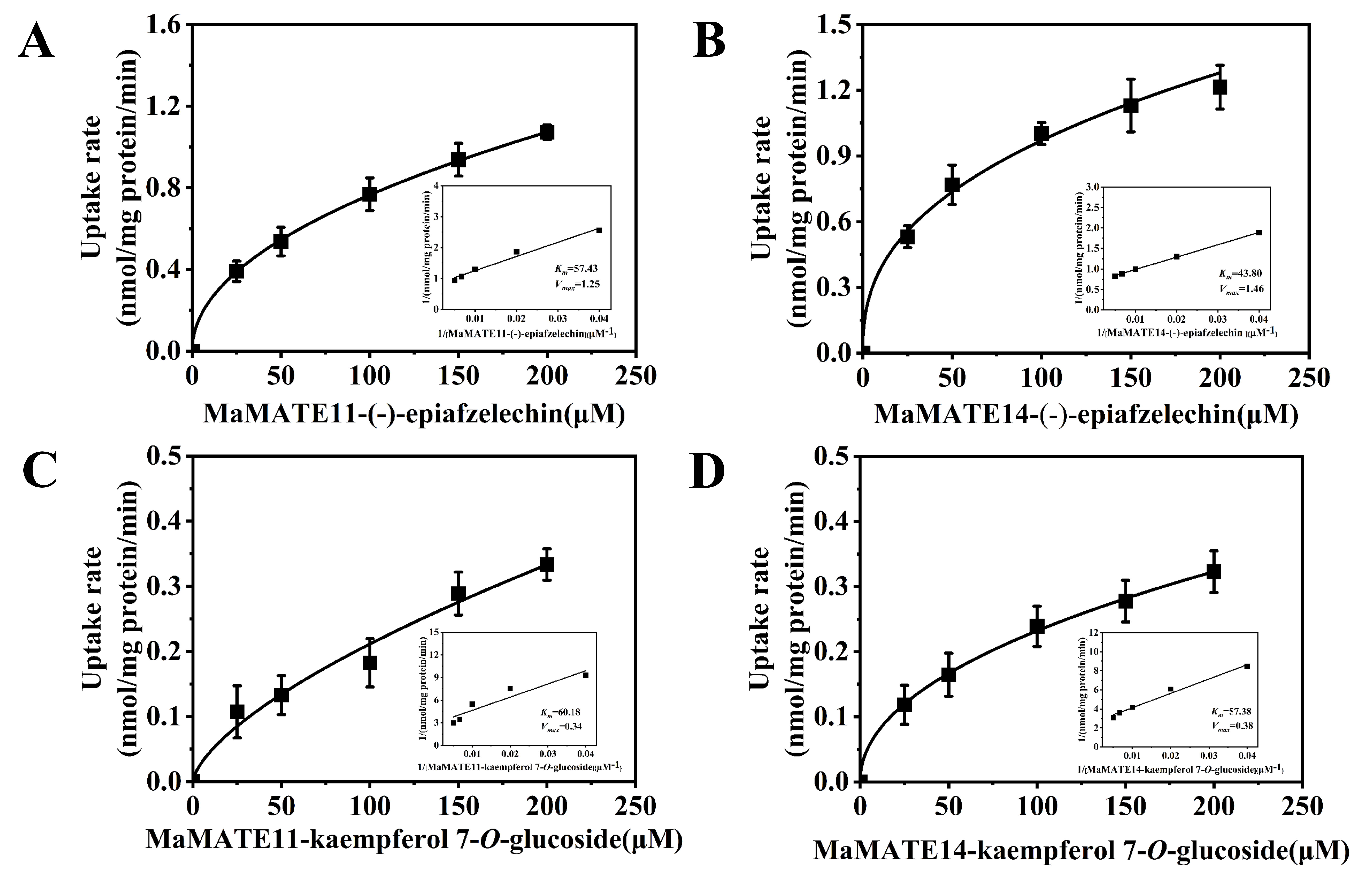


**Fig. S9.** Kinetics of uptake of (-)-epiafzelechin and kaempferol 7-*O*-glucoside by yeast microsomal vesicles expressing MaMATE11 and MaMATE14. (A) and (C) Concentration dependence of uptake of (-)-epiafzelechin and kaempferol 7-*O*-glucoside into vesicles from yeast expressing MaMATE11. Double reciprocal plots of initial rate data at different concentrations of (-)-epiafzelechin and kaempferol 7-*O*-glucoside by MaMATE11. (B) and (D) Concentration dependence of uptake of (-)-epiafzelechin and kaempferol 7-*O*-glucoside into vesicles from yeast expressing MaMATE14. Double reciprocal plots of initial rate data at different concentrations of (-)-epiafzelechin and kaempferol 7-*O*-glucoside by MaMATE14. Results are presented as net transport, calculated by subtracting the values measured in the absence of ATP from the values measured in the presence of ATP after 20 min. Data are mean ± standard deviation (SD) in three replicate uptake assays from three independent membrane preparations.


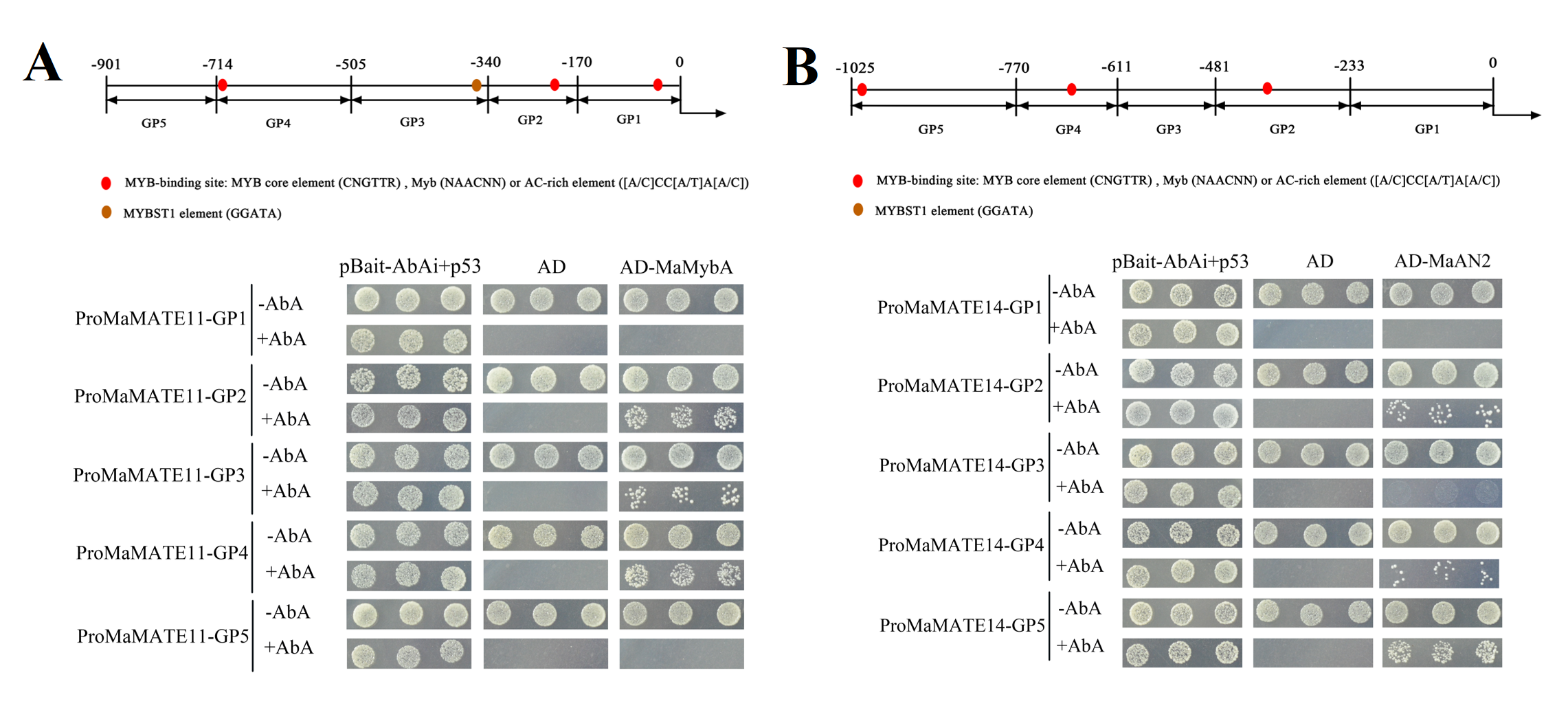


**Fig. S10.** The five fragments (GP1 to GP5) of the promoters of (A) *MaMATE11* and (B) *MaMATE14* and the interaction between them and MaMybA/MaAN2 by Y1H assay, respectively. *ProMaMATE11* and *ProMaMATE14* were divided into GP1 to GP5 relative to the MBS elements. Different MYB protein binding sites (MBS) are marked by red or dark red oval.


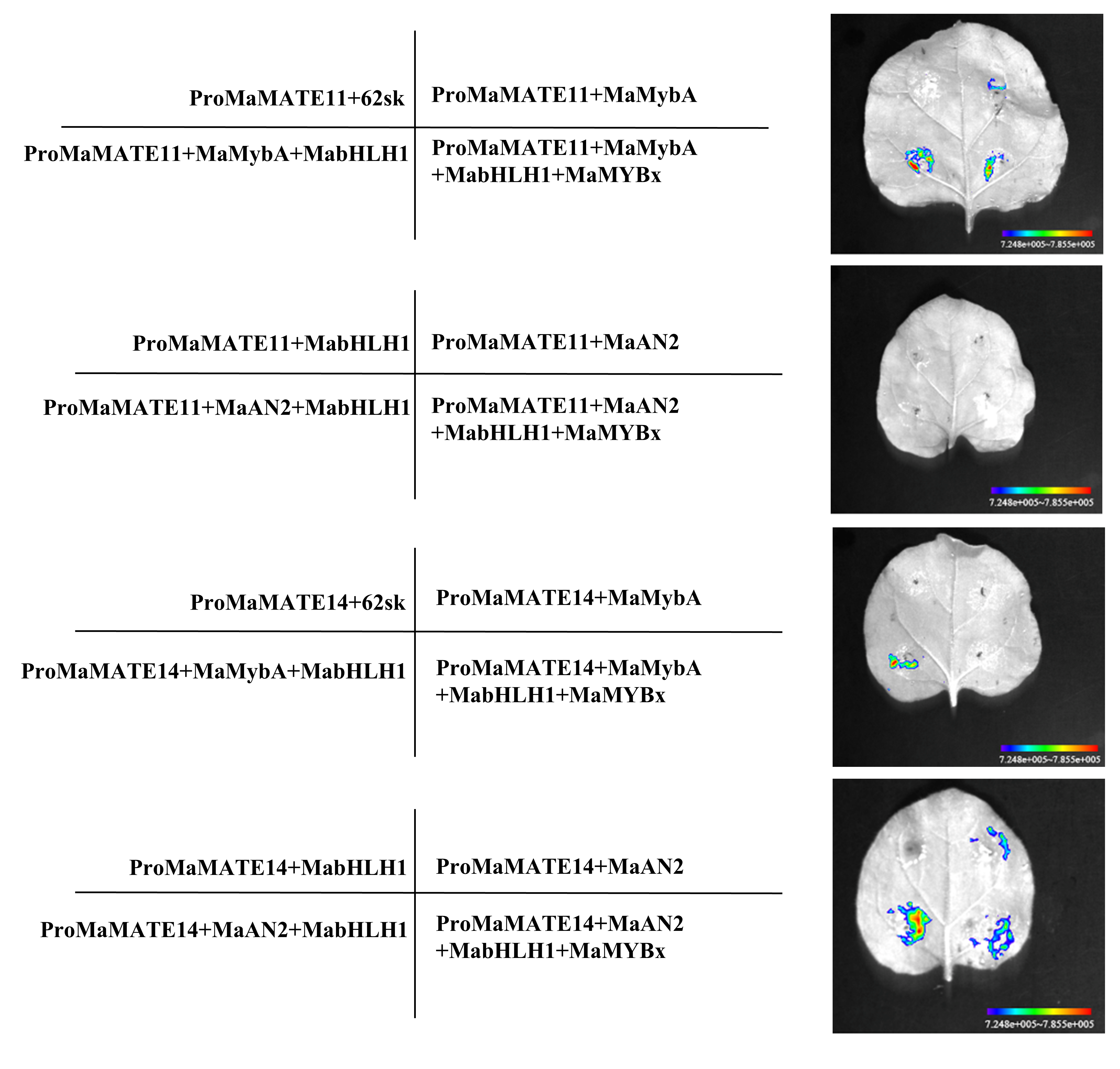


**Fig. S11.**  Fluorescence observations of tobacco leaves.


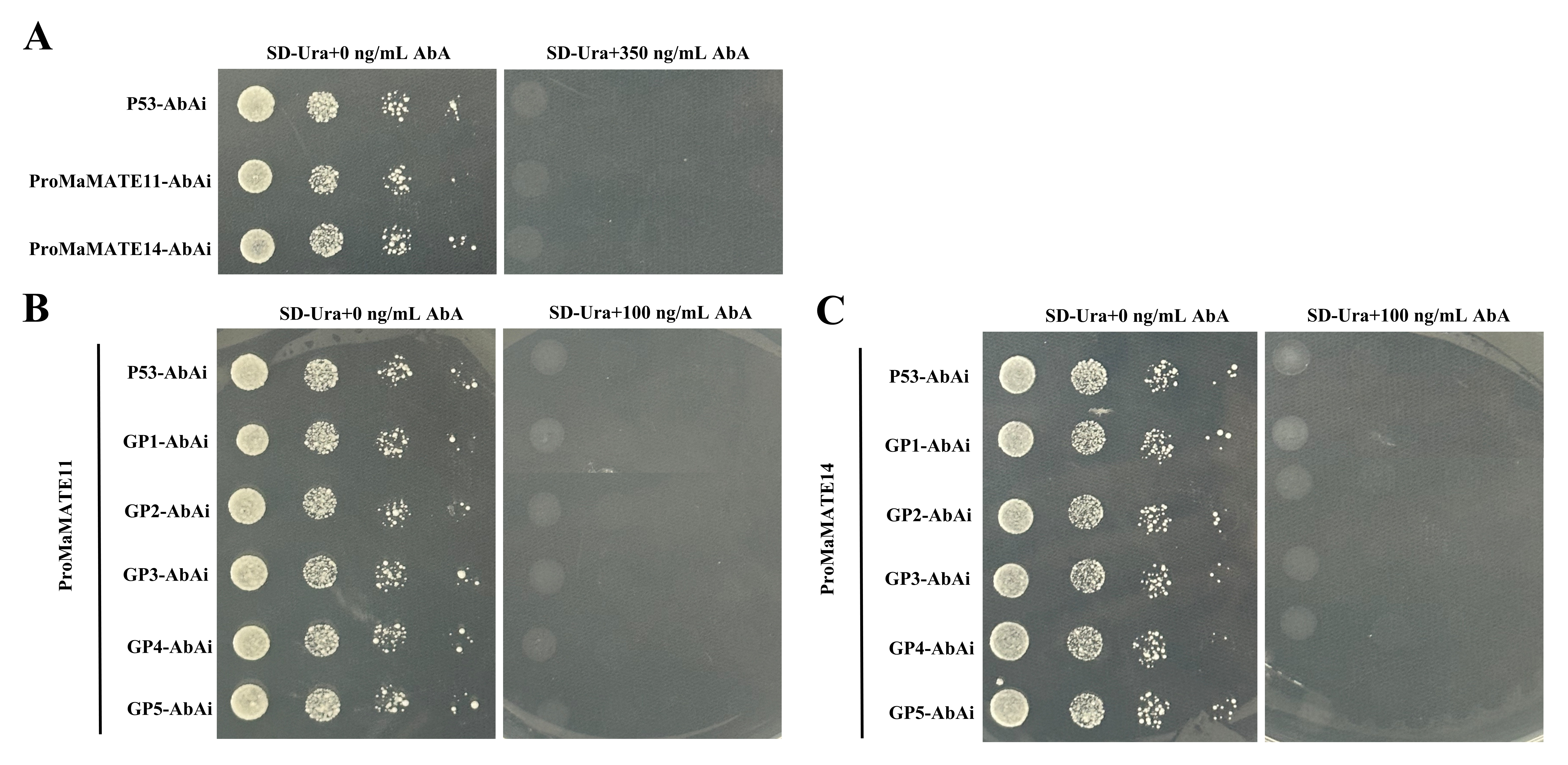


**Fig. S12.** Determine the minimal inhibitory concentration of Aureobasidin A (AbAi) that suppress the self-activation control of *ProMaMATE11/14* (A) and five fragments (GP1 to GP5) of them (B and C).
